# Vaccinia E5 is a major inhibitor of the DNA sensor cGAS

**DOI:** 10.1038/s41467-023-38514-5

**Published:** 2023-05-22

**Authors:** Ning Yang, Yi Wang, Peihong Dai, Tuo Li, Christian Zierhut, Adrian Tan, Tuo Zhang, Jenny Zhaoying Xiang, Alban Ordureau, Hironori Funabiki, Zhijian Chen, Liang Deng

**Affiliations:** 1 Dermatology Service, Department of Medicine, Memorial Sloan Kettering Cancer Center, New York, NY 10065 USA; 2 Department of Molecular Biology, University of Texas Southwestern Medical Center, Dallas, TX 75390 USA; 3 Laboratory of Chromosome and Cell Biology, The Rockefeller University, New York, NY 10065 USA; 4 Genomic Resources Core Facility, Weill Cornell Medical College, New York, NY 10065 USA; 5 Cell Biology Program, Sloan Kettering Institute, Memorial Sloan Kettering Cancer Center, New York, NY 10065 USA; 6 Human Oncology and Pathogenesis Program, Memorial Sloan Kettering Cancer Center, New York, NY 10065 USA; 7 Weill Cornell Medical College, New York, NY 10065 USA; 8 Present Address: The Institute of Cancer Research, London, SW3 6JB UK

**Keywords:** Pox virus, Immune evasion, Viral infection

## Abstract

The DNA sensor cyclic GMP-AMP synthase (cGAS) is critical in host antiviral immunity. Vaccinia virus (VACV) is a large cytoplasmic DNA virus that belongs to the poxvirus family. How vaccinia virus antagonizes the cGAS-mediated cytosolic DNA-sensing pathway is not well understood. In this study, we screened 80 vaccinia genes to identify potential viral inhibitors of the cGAS/Stimulator of interferon gene (STING) pathway. We discovered that vaccinia E5 is a virulence factor and a major inhibitor of cGAS. E5 is responsible for abolishing cGAMP production during vaccinia virus (Western Reserve strain) infection of dendritic cells. E5 localizes to the cytoplasm and nucleus of infected cells. Cytosolic E5 triggers ubiquitination of cGAS and proteasome-dependent degradation via interacting with cGAS. Deleting the E5R gene from the Modified vaccinia virus Ankara (MVA) genome strongly induces type I IFN production by dendritic cells (DCs) and promotes DC maturation, and thereby improves antigen-specific T cell responses.

## Abstract (web summary)

The cGAS-STING signalling pathway is critical in mediating host antiviral immunity. Here, Yang et al screen vaccinia viral genes to identify and then characterise that the viral protein E5 is a major inhibitor of cGAS by mediating cGAS ubiquitination and degradation.

## Introduction

Cyclic GMP-AMP synthase (cGAS) is a major DNA sensor critical to antiviral, antitumor innate immunity, as well as in autoimmune inflammatory diseases^1–4^. Once activated by cytosolic DNA, cGAS generates cyclic GMP-AMP (cGAMP), which in turn binds to an endoplasmic reticulum-localized protein STING, resulting in the activation of the TBK1/IRF3/IFNB pathway. Consequently, viruses have evolved to employ many strategies to evade this important antiviral pathway^5–8^.

Poxviruses are large cytoplasmic DNA viruses that are important human and veterinary pathogens, as well as oncolytic agents and viral vectors^9–11^. Vaccinia virus (VACV) was used successfully as a vaccine for smallpox eradication. However, direct infection of dendritic cells (DCs) with vaccinia results in the inhibition of both innate and adaptive immune responses^12–14^. Modified vaccinia virus Ankara (MVA) is a highly attenuated vaccinia strain with deletion of large fragments from its parental vaccinia genome following more than 570 serial passages in chicken embryo fibroblasts, rendering it non-replicative in most mammalian cells^15,16^. MVA is an important vaccine vector and was recently approved as a second-generation vaccine against smallpox and monkeypox^10,17^.

cGAS is important for host defense against poxvirus infection. cGAS-deficient mice are more susceptible to intranasal infection with VACV^2^ and to footpad inoculation with ectromelia virus, a mouse-specific poxvirus^18^. Vaccinia B2R gene was recently discovered to encode a cytosolic cGAMP nuclease (renamed as poxin), and deletion of B2R from VACV resulted in attenuation in the skin scarification (SS) model^19^. However, whether poxviruses encode a direct inhibitor(s) of cGAS remains unknown.

In this study, we performed a screen of 80 vaccinia viral genes for inhibition of the cGAS/STING pathway using a dual-luciferase reporter assay and identified several vaccinia genes encoding proteins involved in down-regulating the cGAS/STING/IFNB pathway. Here we show that E5 (encoded by the E5R gene), a BEN-domain-containing protein conserved among orthopoxviruses^20^, is a virulence factor and a major inhibitor of cGAS. E5 interacts with cytoplasmic cGAS and triggers its degradation in a proteasome-dependent manner.

## Results

### Screening strategy for identifying viral inhibitors of the cGAS/STING pathway

**Fig. 1:**
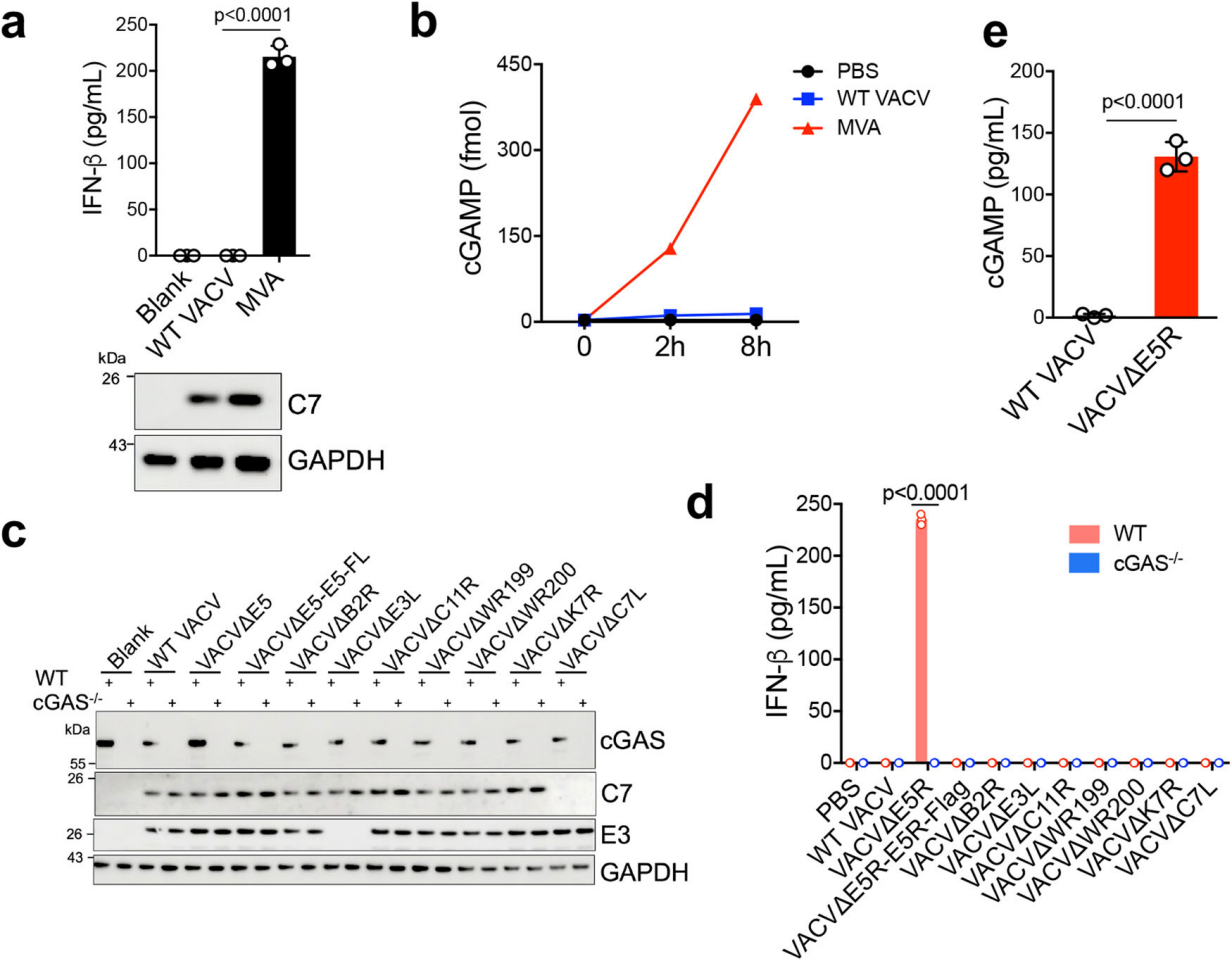
Discovery of vaccinia virus E5 as a key inhibitor of the cGAS-dependent type I IFN pathway. **a** ELISA analysis of IFN-β levels in the supernatants of BMDCs infected with either WT VACV or MVA at an MOI of 10 for 16 h. Cell lysates were analyzed by immunoblot. *n* = 3 independent samples. **b** cGAMP levels in BMDCs infected with either WT VACV or MVA at an MOI of 10. Cells were harvested at 2 and 8 h post-infection. cGAMP levels were measured by LC-MS. **c** Immunoblot of cGAS, C7, and E3 in BMDCs from WT or *cGas*^−/−^ mice infected with different vaccinia viruses at MOI of 10 for 16 h. **d** ELISA analysis of IFN-β levels in BMDCs from WT or *cGas*^−/−^ mice infected with different vaccinia viruses at MOI of 10 for 16 h. *n* = 3 independent samples. **e** ELISA analysis of cGAMP levels in WT BMDCs infected with either WT VACV or VACVΔE5R at MOI of 10 for 16 h. *n* = 3 independent samples. Two-tailed unpaired Student’s *t*-test was used for comparisons of two groups in the studies. Data are representative of two independent experiments and presented as mean ± SEM. Source data are provided as a Source Data file.

Vaccinia virus is a large cytoplasmic DNA virus with a 190 kilobase pairs (kbp) genome that encodes over 200 proteins^21,22^. MVA has an approximately 30-kbp deletion from its parental vaccinia genome, resulting in the loss of many immune-modulatory viral genes^15^. MVA infection of BMDCs induced IFN-β secretion and cGAMP production, whereas wild-type vaccinia strain WR (WT VACV) infection failed to do so (Fig. 1a, b). These results suggest that WT VACV might encode a viral inhibitor(s) to block cGAS activation and downstream IFN-β production.

To identify potential cGAS inhibitors from the vaccinia genome, we first selected 80 viral genes, which include mainly the ones expressed at early times during vaccinia infection^21^, with the reasoning that antagonists of innate immunity are likely encoded by viral early genes. A dual-luciferase assay was then performed to screen for the abilities of these viral proteins to inhibit the cGAS/STING-mediated cytosolic DNA-sensing pathway. Similar strategies have been successfully used to screen for inhibitors of this pathway by other DNA viruses, as previously reported^5,23^. In our system, transfection of HEK293 T cells with 10 ng of STING had limited IFNB promoter induction effect, whereas transfection with 25 ng STING enhanced IFNB-firefly luciferase signal, suggesting a high amount STING alone without cGAS could produce luciferase signal (Supplementary Fig. 1a). Therefore, we used co-transfection of STING (10 ng) and cGAS (50 ng) in our screen with the intention to select potential cGAS inhibitors (Supplementary Fig. 1b, c). Adenovirus E1A encodes a known inhibitor of STING^6^, and the vaccinia C7L gene was recently reported by our group to encode an inhibitor of the IRF3/IFNB pathway by preventing IRF3 phosphorylation^24^. Here we showed that co-transfection of E1A or C7L with STING (10 ng) and cGAS (50 ng) resulted in the inhibition of IFNB-firefly luciferase signal (Supplementary Fig. 1b). Therefore, they were selected as positive controls for the screen. For the actual screen, HEK293T cells were transfected with plasmids expressing an IFNB-firefly luciferase reporter, a pRL-TK control plasmid expressing *Renilla* luciferase, cGAS (50 ng), STING (10 ng), and individual vaccinia viral genes as indicated, as well as E1A and C7R (Supplementary Fig. 1c). This assay identified several vaccinia viral early genes (E5R, K7R, B14R, C11R, WR199/B18R, WR200/B19R, E4L) as potential inhibitors of the cGAS/STING pathway (Supplementary Fig. 1c, d). To test whether any of the candidate inhibitors attenuate STING-mediated IFNB promoter activation, we co-transfected individual candidate genes together with STING at a higher amount (50 ng), which causes induction of IFNB promoter without cGAS. We found that over-expression of all of the candidates except B14R had little effect on STING (50 ng)-induced IFNB promoter activity, suggesting that B14 might target STING or its downstream signaling pathways while other candidates might target cGAS (Supplementary Fig. 1e). Among these genes, WR200/B19R is known to encode a type I IFN binding protein^25^. Although K7, B14, and C11 have been described as vaccinia virulence factors^26–28^, and WR199/B18R was reported to encode a host range factor^29,30^, how they evade the type I IFN pathway is unclear. E4L encodes vaccinia RNA polymerase subunit RPO30 and an intermediate transcription factor^31,32^. While E5 was reported to be a viral early protein associated with the virosomes (viral factories)^33^, whether or not it plays a role in immune evasion is unknown.

### Deleting the E5R gene from the WT VACV genome results in cGAS-dependent type I IFN induction in DCs

We hypothesized that deleting a major inhibitor of the cGAS/STING pathway from the VACV genome would result in higher induction of type I IFN than other deletion mutants or the parental virus. To test this idea, we generated a series of recombinant VACV viruses with deletions of individual candidate viral inhibitors, including VACV∆E5R, VACV∆B2R, VACV∆E3L, VACV∆C11R, VACV∆WR199, VACV∆WR200, VACV∆K7R, and VACV∆C7L. WT and cGAS^−/−^ BMDCs were infected with WT VACV and the deletion mutants. The expression of viral proteins C7 and E3 was determined to verify viral infection (Fig. 1c). IFN-β concentrations in the supernatants of infected BMDCs were determined by ELISA. WT VACV infection of BMDCs failed to induce IFN-β production. Among all of the deletion mutants, only VACV∆E5R induced IFN-β secretion from WT BMDCs, but not from cGAS^−/−^ BMDCs (Fig. 1d). VACV∆E5R-E5R-Flag in which E5R was replaced by E5R-Flag failed to induce IFN-β secretion, signifying that E5R-Flag is biologically active (Fig. 1d). Moreover, although B2 (encoded by the B2R gene) was identified as a cGAMP nuclease^19^, VACV∆B2R infection did not induce IFN-β secretion from WT BMDCs (Fig. 1d), suggesting the presence of other viral inhibitors of the cGAS/STING/IFNB pathway in VACV∆B2R. In addition, VACV∆E5R infection of WT BMDCs induced cGAMP production, indicating cGAS activation (Fig. 1e). These results demonstrate that vaccinia E5R encodes a major inhibitor of cGAS.

### The vaccinia E5R gene, which encodes a BEN-domain protein, is conserved among orthopoxviruses

Vaccinia E5 is a 341-amino acid polypeptide comprising an N-terminal alpha-helical domain (amino acids 60–106) and two BEN domains at the C-terminus (amino acid 112–222 and amino acid 233–328) (Supplementary Fig. 2a). BEN was named for its presence in BANP/SMAR1, poxvirus E5R, and NAC1^20^, and BEN domain-containing proteins function in DNA binding, chromatin organization, and transcriptional repression^34–36^. E5R is conserved among orthopoxviruses (Supplementary Fig. 2b. c) but less so among yatapoxviruses and myxoma virus. However, E5R orthologs are absent in parapoxviruses, entomopoxviruses, fowlpox, and molluscum contagiosum viruses (data not shown). The E5 proteins from vaccinia (Western Reserve (WR) strain and Copenhagen strain), variola (the causative agent for smallpox), and cowpox viruses contain an extra 10-amino acid sequence at the N-termini compared with E5 proteins from vaccinia (Ankara), MVA, and ectromelia (mousepox) (Supplementary Fig. 2c). Interestingly, Monkeypox E5 has large deletions at both its N- and C-termini (Supplementary Fig. 2c)^37^.

### Vaccinia virus E5 is a virulence factor

**Fig. 2:**
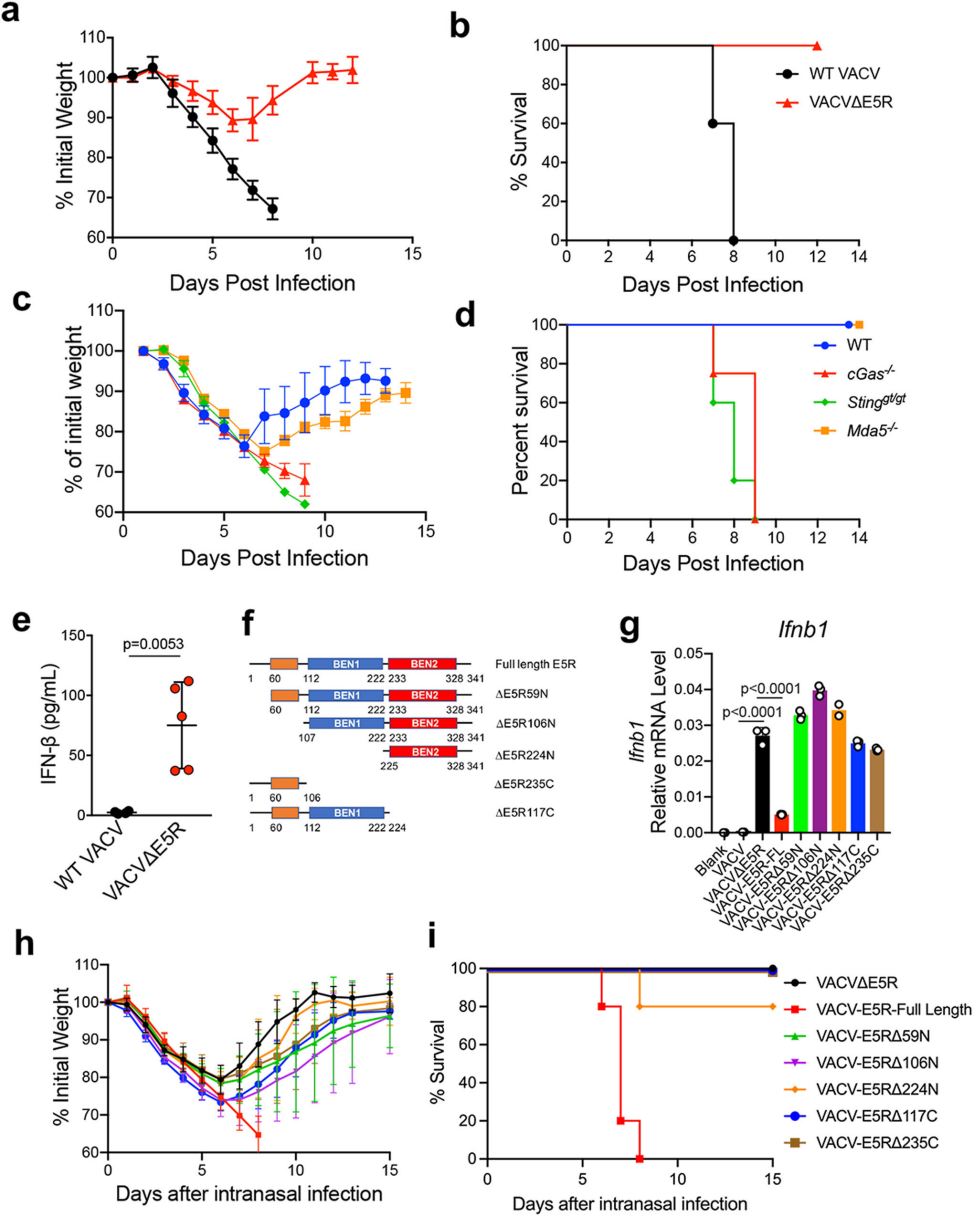
Vaccinia virus E5 is a virulence factor in vivo. **a,** **b** Percentages of initial weight (**a**) and Kaplan–Meier survival curves (**b**) of WT C57BL/6J mice (*n* = 5 in each group) over days post-intranasal infection with either WT VACV or VACV∆E5R at a dose of 2 × 10^6^ pfu per mouse. **c**, **d** Percentages of initial weight (**c**) and Kaplan–Meier survival curves (**d**) of WT, *Mda5*^−/−^, *cGas*^−/−^, or *Sting*^*gt/gt*^ mice (*n* = 5 in each group) over days post-intranasal infection with VACV∆E5R at a dose of 2 × 10^7^ pfu per mouse. **e** ELISA analysis of IFN-β levels in the BALF harvested from WT mice at 48 h post-infection with WT VACV or VACV∆E5R at a dose of 2 × 10^7^ pfu per mouse. *n* = 5 independent samples. **f** Schematic diagram of VACV E5R full-length revertant and various VACV E5R truncation mutants. **g** RT-qPCR analysis of *Ifnb* levels in WT BMDCs infected with different vaccinia viruses, including VACV, VACV∆E5R, VACV-E5R-FL revertant, and various VACV E5R truncation mutants at MOI of 10 for 6 h. *n* = 3 independent samples. **h**, **i** Percentages of initial weight (**h**) and Kaplan–Meier survival curves (**i**) of WT C57BL/6J mice (*n* = 5 in each group) over days post-intranasal infection with VACV∆E5R, VACV-E5R-full length revertant, and various E5R truncation mutants a dose of 2 × 10^7^ pfu per mouse. Two-tailed unpaired Student’s *t-*test was used for comparisons of two groups in the studies. Data are presented as mean ± SEM. Source data are provided as a Source Data file.

To test whether vaccinia E5 is a virulence factor, we performed an intranasal infection experiment with WT VACV or VACV∆E5R (2 × 10^6^ pfu) in WT C57BL/6J mice. All of the mice infected with WT VACV lost weight quickly, starting on the third day of infection, and either died or were euthanized due to more than 30% weight loss at days 7 to 8 post-infection (Fig. 2a, b). By contrast, mice infected with VACV∆E5R lost close to 15% of initial body weight on average at day 6 post-infection and then recovered (Fig. 2a, b). To rule out the possibility that VACV∆E5R has attenuation in replication, we compared the plaque sizes and replication kinetics between WT VACV and VACV∆E5R in BSC40 cells. We found that VACV∆E5R was replication-competent and exhibited similar replication kinetics as WT VACV in vitro (Supplementary Fig. 3a, b). However, intranasal infection of WT VACV at 2 × 10^5^ pfu resulted in much higher titers in the infected lungs, spleens, livers, and blood at day 6 post-infection compared with those tissues harvested from VACV∆E5R (2 × 10^7^ pfu)-infected C57BL/6J mice at day 6 post-infection (Supplementary Fig. 3c). These results demonstrate that although VACV∆E5R has similar replication capacity as WT VACV in vitro, it is attenuated in vivo, likely due to host innate immune responses induced by VACV∆E5R infection. The reduced viral titers in various tissues after VACV∆E5R infection correlates with less weight loss and better survival of the infected animals (Fig. 2a, b). These results indicate that VACV∆E5R is attenuated compared with WT VACV and thereby demonstrate that E5 is a virulence factor. Furthermore, VACV∆E5R (2 × 10^7^ pfu) gained virulence in *cGas*^−/−^, or *Sting*^*gt/gt*^ mice but remained attenuated in *Mda5*^−/−^ mice, indicating the cytosolic DNA-sensing pathway mediated by cGAS or STING is indispensable for host defense against intranasal infection with VACV∆E5R (Fig. 2c, d). Moreover, we detected IFN-β in the bronchoalveolar lavage (BALF) 48 h after VACV∆E5R infection (Fig. 2e), indicating that intranasal infection with VACV∆E5R infection could induce IFN-β production in vivo.

To determine which domain(s) of E5 are required for E5-mediated inhibition of IFNB induction and virulence, we constructed VACV-E5R (full-length; FL) and a series of its truncation mutants (Fig. 2f). BMDCs were infected with WT VACV, VACV∆E5R, VACV-E5R-FL, and the E5R truncation mutants. Vaccinia C7 expression was determined by Western blot analysis to verify viral infection (Supplementary Fig. 3d). Quantitative real-time PCR was used to determine *Ifnb1* gene expression in infected BMDCs. Whereas VACV-E5R (full-length) only mildly induced *Ifnb1* gene expression in BMDC cells (Fig. 2g), all E5R truncation mutants induced *Ifnb1* gene expression at a similar level to VACV∆E5R (Fig. 2g). Moreover, intranasal infection of VACV-E5R (full-length) and E5R truncation mutants (2 × 10^7^ pfu) in C57BL/6J mice showed that only VACV-E5R (full-length) infection was lethal, while all of the truncation mutants caused transient weight loss but 100% survival, except for VACV-E5R∆224N (with 80% survival) (Fig. 2h, i). These results demonstrated that all E5 domains, including the N-terminal alpha-helical domain and the two BEN domains, are required for the repression of Ifnb gene expression and virulence.

### Deleting E5R from the MVA genome strongly induces cGAMP production and type I IFN secretion in BMDCs

**Fig. 3:**
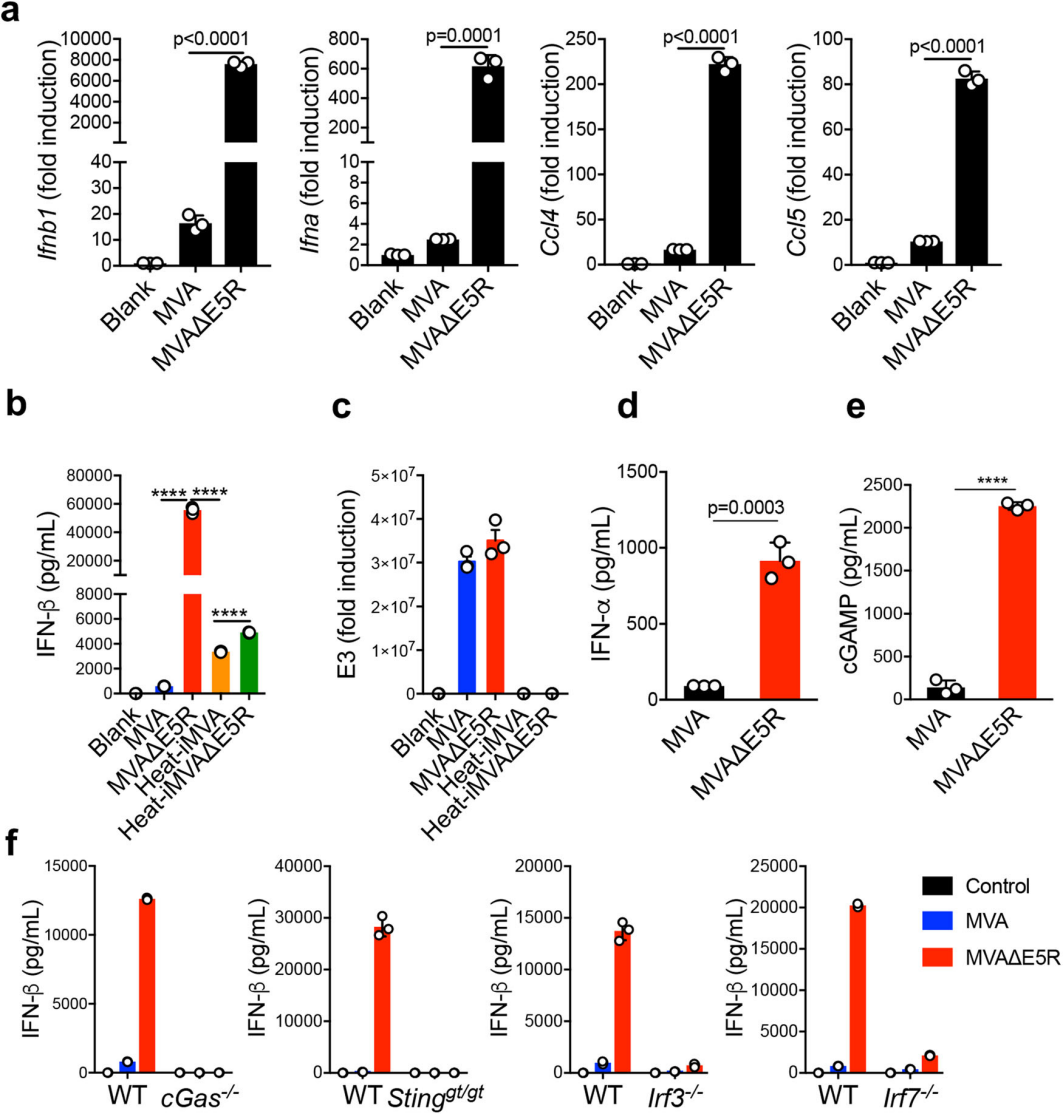
MVA∆E5R strongly induces type I IFN production in a cGAS/STING, IRF3/IRF7-dependent manner. **a** RT-qPCR of *Ifnb1*, *Ifna*, *Ccl4*, and *Ccl5* gene expression in WT BMDCs infected with either MVA or MVA∆E5R at an MOI of 10 for 6 h. *n* = 3 independent samples. **b** ELISA analyses of IFN-β or IFN-α levels in the supernatants of WT BMDCs infected with MVA, MVA∆E5R, Heat-iMVA, or Heat-iMVA∆E5R at an MOI of 10 for 16 h. *n* = 3 independent samples. **c** RT-qPCR analysis of E3 gene expression in WT BMDCs infected with different vaccinia viruses at MOI of 10 for 16 h. *n* = 3 independent samples. **d** ELISA analyses of IFN-α levels in the supernatants of WT BMDCs infected with MVA or MVA∆E5R at an MOI of 10 for 16 h. *n* = 3 independent samples. **e** ELISA analyses of cGAMP levels in WT BMDCs infected with MVA or MVA∆E5R at an MOI of 10 for 16 h. *n* = 3 independent samples. **f** ELISA analyses of IFN-β levels in the BMDC from WT, *cGas*^−/−^, *Sting*^*gt/gt*^, *Irf3*^−/−^, and *Irf7*^−/−^ mice infected with MVA or MVA∆E5R at an MOI of 10 for 16 h. *n* = 3 independent samples. ***p < 0.001. Two-tailed unpaired Student’s *t*-test was used for comparisons of two groups in the studies. Data are presented as mean ± SEM. Source data are provided as a Source Data file.

To investigate whether the E5R gene of the MVA genome encodes a functional protein, we generated MVA∆E5R. MVA∆E5R infection of BMDCs potently upregulated *Ifnb1*, *Ifna*, *Ccl4*, and *Ccl5* gene expression (Fig. 3a), whereas MVA infection had modest induction. MVA∆E5R infection of BMDCs induced much higher levels of IFN-β secretion than MVA, heat-inactivated MVA (heat-iMVA), or heat-inactivated MVA∆E5R (heat-iMVA∆E5R) (Fig. 3b). Vaccinia E3L expression was comparable between MVA and MVA∆E5R (Fig. 3c). E3L gene expression was not detected in heat-inactivated MVA (Heat-iMVA) or heat-inactivated MVA∆E5R (Heat-iMVA∆E5R)-infected BMDCs as expected. IFN-β induction in BMDCs depended on viral doses (Supplementary Fig. 4a). IFN-α secretion was also strongly induced by MVA∆E5R infection in BMDCs (Fig. 3d). Furthermore, MVA∆E5R caused much higher levels of cGAMP production in BMDCs than MVA (Fig. 3e), suggesting that E5 targets cGAS.

Similar to BMDC, MVA∆E5R infection of bone marrow-derived macrophages (BMM) or primary dermal fibroblasts also induced much higher levels of IFN-β secretion than MVA (Supplementary Fig. 4b, c). These results demonstrated that vaccinia E5 blocks cGAS activation and deletion of E5R from the MVA genome potently activates the cGAS/STING pathway in multiple myeloid cell types. Furthermore, MVA∆E5R-induced IFN-β secretion from BMDCs was abolished in cGAS^−/−^ or STING^Gt/Gt^ cells and diminished in IRF3^−/−^ or IRF7^−/−^ cells (Fig. 3f). In addition to BMDCs, MVA∆E5R-induced IFN-β secretion in BMMs or primary fibroblasts was also dependent on the cGAS/STING pathway (Supplementary Fig. 4b, c). These results demonstrate that the cGAS/STING-mediated cytosolic DNA-sensing pathway and the transcription factor IRF3 and IRF7 are required for MVA∆E5R-induced IFN-β secretion in various primary cell types.

In addition to parental viral DNA provided by the incoming virions, progeny viral DNA generated after DNA replication in the virosomes may also stimulate the cytosolic DNA sensor cGAS, resulting in IFN-β production. To evaluate this, we used phosphonoacetate (PAA) or aphidicolin to block viral DNA replication^38,39^. PAA or aphidicolin treatment of MVA∆E5R-infected MEFs blocked virosome formation as expected (Supplementary Fig. 4d) and abolished the expressions of viral late genes such as A27 and A34 (Supplementary Fig. 4e). Both *Ifnb1* and *Ifna* gene expressions induced by MVA∆E5R were partially reduced in the presence of PAA or aphidicolin (Supplementary Fig. 2e). Overall, our results indicate both the parental and progeny viral DNA from MVA∆E5R-infected BMDCs contribute to type I IFN induction.

### WT VACV or MVA infection triggers cGAS degradation via a proteasome-dependent mechanism

**Fig. 4:**
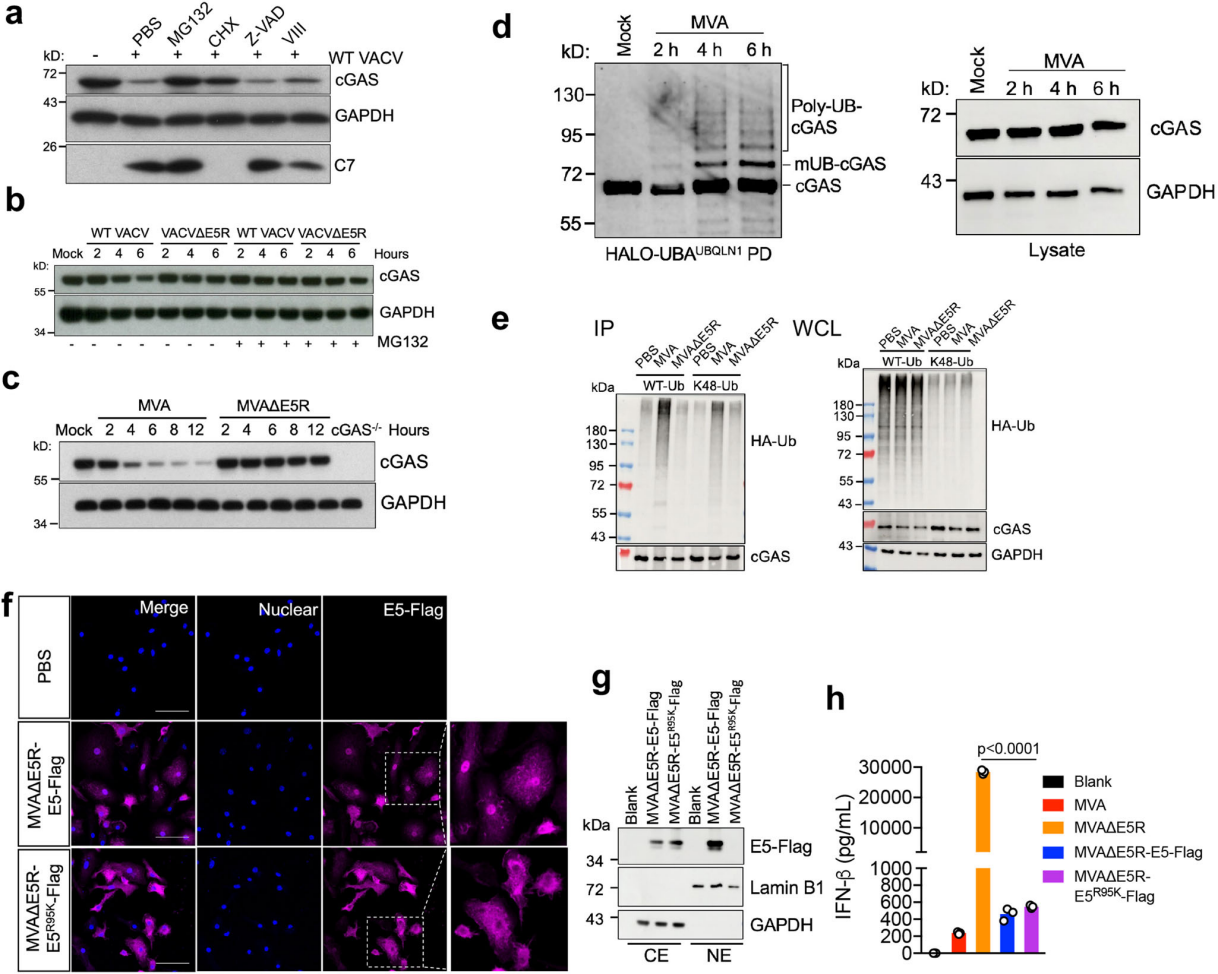
WT VACV or MVA infection induces proteasome-dependent degradation of cGAS. **a** Immunoblot of cGAS in MEFs infected with WT VACV at an MOI of 10 for 6 h. Cells were pretreated with cycloheximide (CHX, 25 µg/mL), proteasome inhibitor MG132 (25 µM), pan-caspase inhibitor Z-VAD (50 µM), AKT1/2 inhibitor VIII (10 µM) for 30 min and then infected with WT VACV in the presence of each individual drug. Cells were collected at 6 h post-infection. **b**, **c** Immunoblot of cGAS in BMDCs infected with the indicated virus at an MOI of 10. Cells were pre-treated with or without MG132 (25 µM) for 30 min before being infected with the virus. Cells were collected at indicated time post-infection. **d** BMDCs were infected with MVA at an MOI 10. At indicated time points, cells were collected, and total ubiquitinated proteins were pulled down by Halo-4xUBA^UBQLN1^ beads. Ubiquitinated cGAS were detected by the anti-cGAS antibody. mUB-cGAS monoubiquitinated cGAS, Poly-UB-cGAS polyubiquitinated cGAS, PD pull-down. **e** HEK293T cells were co-transfected with V5–cGAS and HA-Ub (WT or K48 only)-expressing plasmids. Twenty-four hours later, cells were infected with MVA or MVAΔE5R at MOI 10 for 6 h in the presence of MG132 (25 µM). cGAS was pulled down by an anti-V5 antibody, and ubiquitinated cGAS was determined by an anti-HA antibody. IP immunoprecipitation. WCL whole cell lysates. **f** Representative confocal images showing E5-Flag location in BMDCs after MVAΔE5R-E5-Flag or MVAΔE5R-E5^R95K^-Flag infection at an MOI 10 for 6 h. E5-Flag was stained by an anti-Flag antibody. Scale bar, 50 μm. **g** Immunoblot of E5-Flag in cytoplasmic and nuclear extracts from BMDCs infected with MVAΔE5R-E5-Flag or MVAΔE5R-E5^R95K^-Flag at MOI of 10 for 6 h. CE cytoplasmic extract, NE nuclear extract. **h** ELISA analyses of IFN-β levels in supernatants of BMDCs infected with MVA, MVA∆E5R, MVAΔE5R-E5-Flag or MVAΔE5R- E5^R95K^-Flag at an MOI of 10 for 16 h. *n* = 3 independent samples. Two-tailed unpaired Student’s *t-*test was used for comparisons of two groups in the studies. Data are representative of two (**f**) or three (**a**–**e**) independent experiments and presented as mean ± SEM. Source data are provided as a Source Data file.

To investigate how E5 antagonizes the cGAS/STING pathway, we first evaluated cGAS protein levels after WT VACV infection. We observed that cGAS protein levels were lower at six hours after WT VACV infection in BMDCs compared with mock-infection control (Fig. 4a), suggesting that cGAS protein might be degraded after viral infection. Treatment with proteasome inhibitor MG132 prevented cGAS degradation, whereas treatment with a pan-caspase inhibitor, Z-VAD, or an AKT1/2 inhibitor VIII had little effect on cGAS levels (Fig. 4a). Treatment with the protein translation inhibitor cycloheximide (CHX) partially blocked cGAS degradation, suggesting that the newly synthesized viral proteins might facilitate cGAS degradation (Fig. 4a). Vaccinia C7 protein level in infected cells was used to verify infection and drug effects. For example, C7 protein was absent in cells infected with WT VACV in the presence of CHX (Fig. 4a). These results indicate that WT VACV-induced cGAS degradation is proteasome-dependent. Unlike WT VACV, VACV∆E5R infection of BMDCs did not result in cGAS degradation (Fig. 4b). Similarly, whereas MVA infection of BMDCs triggered cGAS degradation, MVA∆E5R infection did not (Fig. 4c), confirming that E5 contributes to cGAS degradation in the context of either VACV or MVA infection.

To test whether MVA infection of BMDCs triggers ubiquitination of endogenous cGAS, BMDCs were infected with MVA at an MOI of 10 in the presence of MG132 for indicated times. Total ubiquitinated proteins from MVA-infected BMDC were enriched by Halo-4×UBA^UBQLN1^ beads, which contain four tandem ubiquitin-associated (UBA) domains from Ubiquilin-1 to specifically pull down ubiquitinated proteins^40^. Both monoubiquitinated and polyubiquitinated cGAS bands were detected as early as 2 h post-infection and increased at 4 and 6 h post-infection (Fig. 4d). This ubiquitination pattern correlated with the kinetics of cGAS degradation. We also detected unmodified cGAS in all of the samples, which is most likely due to the non-specific binding of unmodified cGAS to the beads and/or the formation of oligomer complexes with ubiquitinated cGAS.

We hypothesized that vaccinia E5 might induce cGAS ubiquitination and subsequent proteasome-dependent degradation. To test that, HEK293T cells were co-transfected with V5–cGAS and HA-Ub (WT or K48 only)-expressing plasmids. Twenty-four hours later, cells were then infected with MVA or MVAΔE5R in the presence of MG132. cGAS was pulled down by an anti-V5 antibody, and ubiquitinated cGAS was determined by an anti-HA antibody. We detected higher levels of cGAS ubiquitination, particularly K48-linked poly-ubiquitination, in cells infected with MVA compared with those infected with MVA∆E5R (Fig. 4e). Thus, our results suggest that E5 expressed by MVA promotes K48-linked poly-ubiquitination of cGAS leading to its degradation.

### E5 is localized to the cytoplasm and nucleus of infected cells, and cytoplasmic E5 is sufficient to suppress type I IFN production

To visualize E5 expression, we generated a recombinant virus MVA∆E5R-E5-Flag. Confocal imaging of BMDCs infected with MVA∆E5R-E5-Flag showed both cytoplasmic and nucleus localization of E5 (Fig. 4f). Western blot analysis confirmed that E5 was expressed in both the cytoplasm and nucleus (Fig. 4g). GAPDH was used as a positive control for cytoplasmic protein and Lamin B1 was used as a positive control for nucleus protein. Furthermore, MVA∆E5R-E5-Flag infection of BMDCs resulted in much lower IFN-β production than MVA∆E5R (Fig. 4h), indicating that tagging of E5 with Flag at the C-terminus of the protein retained its inhibitory function on the cGAS/STING pathway. In the process of generating the MVA∆E5R-E5-Flag virus using drug selection, we isolated one mutant strain, MVA∆E5R-E5^R95K^-Flag, which contains a single nucleotide change (G284A), resulting in the replacement of arginine at amino acid 95 of E5 by lysine. E5^R95K^-Flag was expressed in the cytoplasm but was not in the nucleus of MVA∆E5R-E5^R95K^-Flag-infected BMDCs (Fig. 4f). Western blot confirmed that only cytoplasmic but not nucleus E5^R95K^-Flag protein was detected after MVA∆E5R-E5^R95K^-Flag infection in BMDCs (Fig. 4g). Furthermore, IFN-β production was diminished in BMDCs infected with MVA∆E5R-E5^R95K^-Flag (Fig. 4h), which was similar to that produced by BMDCs infected with MVA∆E5R-E5-FLAG, indicating that the cytoplasmic E5 is sufficient to suppress type I IFN production.

### Vaccinia virus E5 protein interacts with cGAS and promotes ubiquitination of cGAS and subsequent degradation

**Fig. 5:**
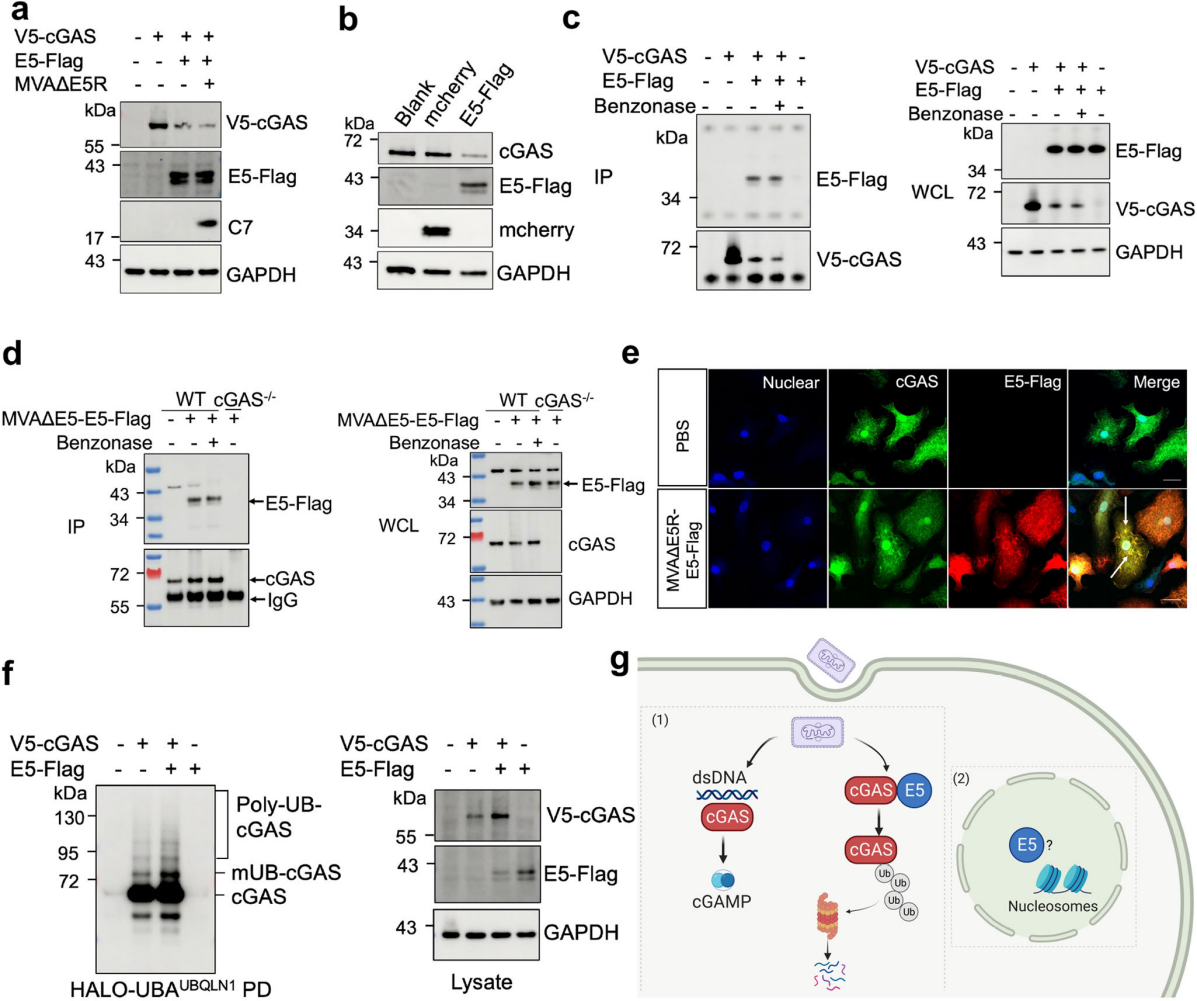
Vaccinia virus E5 interacts with cGAS and promotes cGAS ubiquitination. **a** HEK293T cells were co-transfected with V5–cGAS and pcDNA-E5R-Flag or pcDNA3.1 plasmids. Twenty-four hours later, cells were infected with MVAΔE5R at an MOI of 10 for 6 h. V5–cGAS protein levels were determined by an anti-V5 antibody. C7 protein was measured to indicate MVAΔE5R infection. **b** MEF cells were infected with retrovirus expressing E5R-Flag or mcherry. Forty-eight hours later, cGAS protein levels were determined by an anti-cGAS antibody. **c** HEK293T cells were transfected with an V5–cGAS and pcDNA-E5R-Flag or pcDNA3.1 plasmids. Twenty-four hours later, cell lysates were treated with or without Benzonase (250 U/mL). V5–cGAS was pulled down by an anti-V5 antibody, and E5-Flag was determined by an anti-Flag antibody. **d** BMDCs from WT or *cGas*^−/−^ mice were infected with MVAΔE5R-E5R-Flag at an MOI of 10 for 6 h. Cell lysates were treated with or without Benzonase (250 U/mL). cGAS was pulled down by an anti-cGAS antibody, and E5-Flag was determined by an anti-Flag antibody. **e** Representative confocal images showing E5-Flag and cGAS locations in BMDCs after MVAΔE5R-E5-Flag infection at an MOI 10 for 6 h. E5-Flag was stained by an anti-Flag antibody. Endogenous cGAS was stained by an anti-cGAS antibody. Scale bar, 15 μm. **f** HEK293T cells were transfected with a V5–cGAS and pcDNA-E5R-Flag or pcDNA3.1 plasmids. Twenty-four hours later, cells were collected after being treated with MG132 (25 µM) for 6 h, and total ubiquitinated proteins were pulled down by Halo-4xUBA^UBQLN1^ beads. Ubiquitinated cGAS were detected by the anti-cGAS antibody. mUB-cGAS monoubiquitinated cGAS, Poly-UB-cGAS polyubiquitinated cGAS, PD pull-down. **g** Working model of vaccinia E5 antagonizes cytoplasmic cGAS. Data are representative of two (**e**) or three (**a**–**d**) independent experiments and presented as mean ± SEM. Source data are provided as a Source Data file.

To test whether E5 alone can trigger cGAS degradation without viral infection, we co-transfected a cGAS-expressing plasmid with an E5R-expressing plasmid or empty vector into HEK293T cells. 24 h later, cells were infected with MVAΔE5R at an MOI of 10 for 6 h. We observed that the cGAS level was decreased after co-transfection with the E5R-expressing plasmid but not the empty vector (Fig. 5a). Moreover, MVA∆E5R infection failed to enhance E5-mediated cGAS degradation (Fig. 5a), indicating that E5 alone can trigger cGAS degradation, likely in the context of plasmid transfection. Next, we tested whether E5 overexpression via lentiviral transduction in MEFs also resulted in cGAS degradation. MEFs were transduced with either a lentiviral vector expressing mcherry or E5-Flag. Forty-eight hours later, cell lysates were collected, and cGAS expression was determined. We observed that E5-Flag overexpression also reduced endogenous cGAS levels in MEFs (Fig. 5b).

To assess whether E5 and cGAS interact with each other, we transfected HEK293T cells with V5–cGAS and E5-Flag expression plasmid. Immunoprecipitation with anti-V5 antibody pulled down E5-Flag, thus indicating an E5–cGAS interaction (Fig. 5c). To test whether DNA is required for mediating E5 and cGAS interaction, cell lysates were treated with or without Benzonase, an endonuclease that removes both DNA and RNA. Benzonase treatment failed to disrupt E5–cGAS interaction, indicating that such interaction is not mediated by DNA (Fig. 5c). Next, we tested whether E5-Flag expressed by MVA∆E5R-E5-Flag interacted with endogenous cGAS in BMDCs. Briefly, WT and cGAS^−/−^ BMDCs were infected with MVA∆E5R-E5-Flag at an MOI of 10 for 6 h. Cell lysates were treated with or without Benzonase. cGAS was pulled down by an anti-cGAS antibody, and E5-Flag was determined by an anti-Flag antibody. Co-immunoprecipitation assay showed that in WT BMDCs, cGAS interacted with E5-Flag, and Benzonase treatment failed to disrupt the interaction (Fig. 5d). We failed to detect such interaction in cGAS^−/−^ BMDCs. We also visualized co-localization of E5-Flag and endogenous cGAS in BMDCs infected with MVA∆E5R–E5-Flag (Fig. 5e). Moreover, the co-transfection cGAS and E5R-expressing plasmids into HEK293T cells promoted stronger cGAS ubiquitination than cGAS alone (Fig. 5f). Collectively, using plasmid transfection, lentiviral transduction, and MVA∆E5R–E5-Flag infection approaches in several mammalian cell types, including HEK293T, MEFs, and BMDCs, we demonstrated interactions between E5 and cGAS, which is independent of DNA-binding. Our results also demonstrate that E5 enhances cGAS ubiquitination and subsequent degradation via a proteasome-dependent mechanism (Fig. 5g).

### Deleting the E5R gene from MVA increases antigen-specific T-cell responses after vaccination

**Fig. 6:**
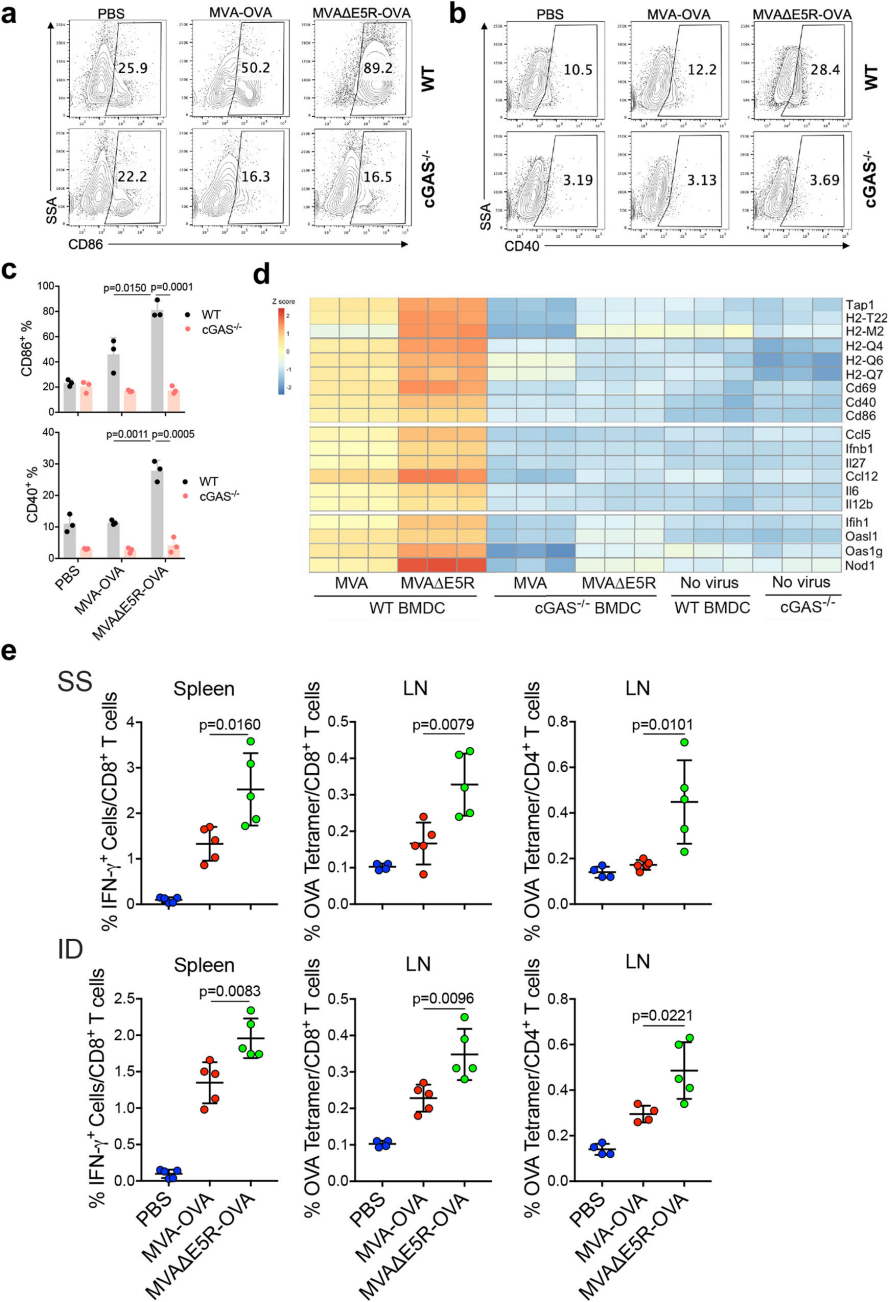
MVA∆E5R promotes dendritic cell maturation and antigen-specific CD8^+^ T cells activation as a vaccine vector. **a**–**c** Representative flow cytometry dot plots (**a,** **b**) or analysis (**c**) of CD86 and CD40 expression in WT or *cGas*^−/−^ BMDC infected with MVA-OVA or MVAΔE5R-OVA at MOI 10 for 16 h. *n* = 3 independent samples. **d** Heatmap showing relative expression of selected immune-related genes in WT or *cGas*^−/−^ BMDC infection with MVA or MVA∆E5R. These include genes involved in antigen presentation, DC activation, IFN and proinflammatory cytokines and chemokines, and innate immune sensors. **e** Antigen-specific T cell responses after vaccination with MVA-OVA or MVA∆E5R-OVA. C57BL/6J mice were vaccinated with MVA-OVA or MVAΔE5R-OVA via skin scarification (SS) or intradermal injection (ID). One week later, spleens and draining lymph nodes (dLNs) were harvested, and SIINFEKL-specific CD8^+^ T cells in splenocytes and OVA tetramer-specific CD8^+^ or Th1 CD4^+^ T cells in lymph nodes were determined by FACS. *n* = 5 independent samples. Two-tailed unpaired Student’s *t*-test was used for comparisons of two groups in the studies. Data are presented as mean ± SEM. Source data are provided as a Source Data file.

MVA has been investigated as a vaccine vector for various infectious diseases^10^, such as HIV, tuberculosis, Malaria, Ebola, and SARS-CoV-2, and MVA infection modestly activates human monocyte-derived DCs (moDCs)^41^. To investigate whether E5R deletion improves the immunogenicity of the viral vector, we first generated MVA∆E5R-OVA, expressing a model antigen chicken ovalbumin (OVA) and then compared DC maturation upon MVA∆E5R-OVA vs. MVA-OVA infection. We observed that MVA∆E5R-OVA infection induced higher levels of CD86 and CD40 expression compared with MVA-OVA at 24 h post-infection (Fig. 6a–c). However, both MVA∆E5R-OVA and MVA-OVA-induced CD86 and CD40 expression diminished in cGAS^−/−^ BMDCs, indicating that the cytosolic DNA-sensing pathway is essential for MVA∆E5R-OVA or MVA-OVA-induced DC maturation (Fig. 6a–c).

RNA-seq analysis of WT or cGAS^−/−^ BMDCs infected or mock-infected with either MVA or MVA∆E5R demonstrated that MVA∆E5R infection in WT BMDCs induced higher levels of type I IFN and pro-inflammatory cytokines and chemokines genes, including *Ifnb1*, *Ccl5*, *Ccl12*, *Il12b*, *Il6*, *Il27*, DC maturation and activation markers such as *CD86*, *CD40*, and *CD69*, as well as genes involved in antigen cross-presentation, including *Tap1*, *H2-Q4*, *H2-Q6*, and *H2-Q7*, compared with MVA (Fig. 6d and Supplementary Fig. 5a, b). The upregulation of these genes by both MVA and MVA∆E5R was cGAS-dependent (Fig. 6d and Supplementary Fig. 5a, c).

Next, we performed vaccination through SS or intradermal (ID) injection with either MVA-OVA or MVA∆E5R-OVA. One week after vaccination, anti-OVA CD8^+^ and Th1 CD4^+^ T cells in the spleens and draining lymph nodes were analyzed. Vaccination with MVA∆E5R-OVA resulted in more OVA-specific CD8^+^ T cells in the spleens than MVA-OVA (Fig. 6e). And more OVA-specific CD8^+^ or Th1 CD4^+^ T cells were detected in the draining lymph nodes (dLN) after MVA∆E5R-OVA vaccination, compared with MVA-OVA (Fig. 6e). These results provide evidence that deletion of the E5R gene from MVA increases antigen-specific T cell responses after vaccinations.

## Discussion

The identification of vaccinia E5 as a major inhibitor of the cytosolic DNA-sensor cGAS highlights the importance of that pathway in host defense against poxvirus infection. E5, a founding member of the BEN-domain family, is conserved among orthopoxviruses. Here we show that the virulence factor E5 binds to cGAS, triggering cGAS ubiquitination and proteasome-dependent degradation and that deleting E5R from the MVA viral vector improves its immunogenicity.

Virulent poxviruses, including VACV (WR and Copenhagen strains), cowpox, and ectromelia virus, fail to activate STING, unlike the highly attenuated derivative, MVA^42,43^. In addition, MVA but not WT VACV infection of BMDCs induces cGAMP production, suggesting that VACV encodes an inhibitor(s) of cGAS. Through an unbiased screen of 80 vaccinia genes, we identified several candidate genes that might encode cGAS inhibitors, including E5R, K7R, C11R, WR199/B18R, and WR200/B19R. We then focused on E5R because a VACV mutant lacking E5R induced a moderate amount of IFN-β secretion and cGAMP production in BMDCs, while VACV mutants lacking other individual candidate genes (including B2R, E3L, C11R, WR199, WR200, K7R, and C7L) failed to induce IFN-β secretion in BMDCs. In our conditions, VACV lacking the B2R gene, which encodes a cGAMP nuclease, fails to induce type I IFN production in BMDC, suggesting that there might be an additional vaccinia viral protein(s) antagonizing the cGAS/STING pathway.

Unlike wild-type VACV, MVA infection of bone marrow-derived dendritic cells induce type I IFN in a cGAS/STING-dependent manner^43^. Our study showed that E5 in MVA is still functional because removing the E5R gene from MVA dramatically enhances type I IFN induction in BMDCs, BMMs, and primary fibroblasts. On the other hand, the presence of E5 in MVA fails to completely block IFN production, which could be attributed to the lack of functional WR200/B19R (IFNAR), B2R, or other potential inhibitors^15^. Previous studies have demonstrated that multiple vaccinia viral proteins target DNA-driven type I IFN induction, such as B2, C4, C16, F17, N2, and C6^19,44–49^. Specifically, C4 and C16 target another DNA sensor, DNA-PK, which play a role in IRF3 activation in fibroblasts^44,45,50^, and both are mutated or lost in MVA^15^.

Although E5 was first identified by mass spectrometry as one of the three major early viral proteins associated with virosomes in vaccinia-infected cells^33^, the function of E5 remained elusive. E5 was also found in the highly purified virions by mass spectrometry after chemical crosslinking, and several interaction partners were identified, including RNA polymerase subunits RAP94, RP147, and NTP1^51^. Here we show E5 presence in both the nucleus and cytoplasm of the infected cells. In MVA∆E5R-E5^R95K^-Flag-infected cells, E5^R95K^ localizes only to the cytoplasm but is sufficient for mediating IFN inhibition. The R95K mutation is within a putative nuclear localization signal of E5, ^92^KFKRMIR^98^.

We show that E5 mediates cGAS degradation via a proteasome-dependent pathway. We propose the following working model based on our results (Fig. 5g). Vaccinia virus enters host cells via macropinocytosis^52^. Upon viral entry, viral DNA is detected by the cytosolic DNA sensor cGAS, whose activation leads to cGAMP production and subsequent STING stimulation. However, in the presence of vaccinia E5, which is mainly synthesized by the incoming virions as an early viral protein, cGAS is targeted for ubiquitination and proteasome-dependent degradation through interacting with E5. This leads to reduced cGAMP production and *Ifnb1* gene expression. It is possible that E5 could be targeted by host proteins such as ISGs. We were not able to show a direct interaction between cGAS and E5 due to the inability to generate recombinant E5 protein despite many attempts in different protein expression systems, including bacterial expression systems using *Escherichia coli*, insect cell expression systems using *Sf9*, and mammalian cell expression systems using Chinese Hamster Ovary. Although we were able to show E5 and cGAS interaction via co-immunoprecipitation in several experimental systems, which was not disrupted by Benzonase treatment, we cannot conclude cGAS and E5 have direct interaction in this study. In addition, we observed that some of the newly expressed E5 translocate to the nucleus. It has been reported that nuclear cGAS is inhibited by nucleosomes^53,54^. The exact functions of nuclear E5 need further investigation.

We previously reported that viral replication is not important for MVA sensing in BMDCs^43^. In this study, however, MVA∆E5R-induced *Ifnb1* gene expression was partially reduced in the presence of the viral DNA replication inhibitors, PAA and aphidicolin. This result suggests that virosomal progeny DNA is detected by cGAS in the setting of MVA∆E5R infection. We surmise that in the presence of E5 during MVA infection, the virosomal progeny DNA might be protected from cGAS sensing.

Various post-translational modifications of cGAS have been reported, including ubiquitination, phosphorylation, acetylation, sumoylation, ISGylation, glutamylation, neddylation, and caspase-mediated cleavage^55,56^. For example, RNF185, a RING domain E3 ubiquitin ligase, has been shown to interact with cGAS during human simplex virus-1 (HSV-1) infection, stimulating K27-linked poly-ubiquitination of cGAS, important for cGAS enzymatic activity^57^. TRIM56, an IFN-inducible E3 ubiquitin ligase, interacts with cGAS to promote monoubiquitination and cGAMP production^58^. In addition, TRIM14, an IFN inducible protein, recruits USP14 to cleave K48-linked poly-ubiquitin chains of cGAS and thereby inhibits cGAS degradation via autophagy^59^. The ubiquitin E3 ligase responsible for K48-linked poly-ubiquitination of cGAS remains elusive^59^. Here we show that MVA infection induces mono-and poly-ubiquitination of cGAS and promotes its degradation in a proteasome-dependent manner. E5 is critical for this process via interacting with cGAS. However, the exact details of how E5 recruits a viral or cellular E3 ubiquitin ligase to catalyze the ubiquitination of cGAS remain to be determined.

The discovery of E5 as a major inhibitor of cGAS provides significant insights into improving MVA as a vaccine vector. Here we show that MVA∆E5R-OVA infection of BMDCs induces high levels of IFN-β production and DC maturation, consistent with cGAS-dependent transcriptomic changes induced by MVA∆E5R. Vaccination with MVA∆E5R-OVA induces higher levels of OVA-specific CD8^+^ and Th1 CD4^+^ T cells compared with MVA-OVA. Recent studies have shown that MVA-based vaccine vectors expressing SARS-CoV-2 spike protein induce potent anti-spike T and B cell immune responses and provide protection in mice, hamsters, and macaques against SARS-CoV-2 infection and lung immunopathology^60–64^. Future investigation of whether MVA∆E5R-based vaccine vectors improve vaccine efficacy against infectious agents such as SARS-CoV-2 is warranted.

Among all of the vaccinia-encoded cGAS inhibitor candidates identified in this study, E5, K7, C11, WR199/B18, and WR200/B19, and previously reported B2 (poxin), only VACV∆E5R infection of BMDCs induced significant levels of type I IFN (Fig. 1d). Given that the activation of the cGAS/STING pathway in DCs is critical for viral-induced oncolytic viral therapy^65^, deleting E5R and perhaps other inhibitors of this pathway from oncolytic vaccinia would improve its safety and therapeutic potential.

## Methods

### Ethics statement

Our research complies with all relevant ethical regulations. We conducted all procedures in strict accordance with the recommendations in the Guide for the Care and Use of Laboratory Animals of the National Institute of Health, and the protocol (#19-01-002) was approved by the Memorial Sloan Kettering Cancer Center (MSKCC) Institutional Animal Care and Use Committee (IACUC). All mice were housed at the MSKCC-specific pathogen-free animal facility under the following conditions: temperatures of 21.1–22.2 °C (70–72 °F), 30–70% humidity, and a 12:12 h light/dark cycle (lights were on from 6 am to 6 pm). Mice were euthanized at the end of the experiment with CO_2_ asphyxiation, with cervical dislocation as a physical secondary assurance method.

### Mice

C57BL/6J mice between 6 and 8 weeks of age were purchased from the Jackson Laboratory and were used for the preparation of bone marrow-derived dendritic cells (BMDCs) and for intranasal infection experiments. *cGas*^−/−^ mice were purchased from the Jackson Laboratory. *Sting*^*gt/gt*^ mice were generated in the laboratory of Russell Vance (University of California, Berkeley)^66^. *Mda5*^−/−^ mice were generated in Marco Colonna’s laboratory (Washington University)^67^. *Irf3*^−/−^ mice were provided by Ruslan Medzhitov (Yale University). *Irf7*^−/−^ mice were generated by Shizuo Akira (Osaka University). All transgenetic mice are C57BJ/6J background.

### Viruses

The WR strain of vaccinia virus (VACV) (ATCC VR-1354) was propagated, and virus titers were determined on BSC40 (African green monkey kidney cells) monolayers at 37 °C. MVA was kindly provided by Gerd Sutter (University of Munich) and propagated in BHK-21 (baby hamster kidney cell, ATCC CCL-10) cells. MVA-OVA was a kind gift from Dr. Ingo Drexler (Heinrich Heine University Dusseldorf). The VACV∆E3L was kindly provided by B. L. Jacobs (Arizona State University). VACV∆C11R virus was kindly provided by Bernard Moss (National Institutes of Health)^68^. All viruses were purified through a 36% sucrose cushion. Heat-iMVA or Heat-iMVA∆E5R was generated by incubating purified MVA or MVA∆E5R virus at 55 °C for 1 hour. To generate recombinant VACV∆E5R virus, BSC40 cells were infected with WT vaccinia virus (WR) at an MOI of 0.2. After 1–2 h, cells were transfected with pE5R-mCherry plasmids with Lipofectamine 2000 (Invitrogen). Homologous recombination between the plasmid DNA and vaccinia viral genome resulted in the deletion of the *E5R* gene from the viral genome and the insertion of mCherry, under the control of the vaccinia synthetic early and late promoter (pSE/L). Cells were collected 2 days later and underwent three cycles of freeze-thaw. Plaque purification was performed based on the mCherry fluorescence seen under the microscope. After 4-5 rounds, pure recombinant VACV∆E5R-mCherry viruses were obtained, and validation of E5R deletion was confirmed by PCR analyses and DNA sequencing. Various other deletion mutants, including VACV∆WR199, VACV∆K7R, VACV∆C7L expressing mcherry, VACV∆B2R, and VACV∆WR200 expressing GFP, were generated following a procedure similar to that described above. VACV-E5R-FL, VACV-E5R∆59N, VACV-E5R∆106N, VACV-E5R∆224N, VACV-E5R∆117C, and VACV-E5R∆235C were generated by inserting full-length or truncated E5R into the E5R locus of VACV∆E5R. MVA∆E5R and MVA∆E5R-OVA expressing mCherry were generated through homologous recombination at the E4L and E6R loci flanking the E5R gene of the MVA or MVA-OVA genome in BHK21 cells following a procedure similar to that described above. MVA∆E5R–E5-Flag was generated by inserting the E5R-Flag sequence into the *TK* locus of MVA∆E5R. MVA∆E5R-E5^R95K^-Flag was generated by inserting the E5R-Flag sequence into the *TK* locus of MVA∆E5R using drug selection. The recombinant virus was enriched in the presence of gpt selection medium, including MPA, xanthine, and hypoxanthine, and plaque was purified for at least four rounds. During the selection process, a spontaneous mutation occurred, resulting in the generation of MVA∆E5R-E5^R95K^-Flag, verified by PCR and DNA sequencing. The recombinant vaccinia viruses were verified by PCR of genomic viral DNA (Supplementary Fig. 7).

### Intranasal infection of vaccinia virus in mice

Female C57BL/6J mice between 6 and 8 weeks of age (5–10 in each group) were anesthetized and infected intranasally with WT VACV, VACV∆E5R or VACV∆E5 expressing E5R full-length revertant and various E5R truncation mutants at the indicated doses in 20 µl PBS. Mice were monitored and weighed daily. Those that lost over 30% of their initial weight were euthanized. Kaplan-Meier survival curves were determined. Bronchoalveolar lavage fluid (BALF) was harvested following intratracheal infusion of 1 mL of cold PBS. To measure viral titers within different organs, lungs, livers, and spleens were harvested, placed into tubes with 1 mL of PBS, and homogenized using the Miltenyi GentleMACS Dissociator. Cell suspensions underwent freeze-thaw cycles three times and sonicated before titration on BSC40 cells. Blood was collected into 1.5 mL Eppendorf tubes, and serum was kept after centrifugation. Virus titers were determined by calculating the number of plaques (pfu) per gram of tissues (pfu/g).

### Multistep growth curve of WT VACV and VACV∆E5R

BSC40 was infected with WT VACV or VACV∆E5R at an MOI of 0.05. The cells were then scraped into the medium and collected at indicated times. After three cycles of freeze-thaw and subsequent sonication, viral titers in the collected samples were determined by plaque assay on BSC40 cells.

### Vaccination with MVA-OVA or MVA∆E5R-OVA

6 to 8-week-old Female C57BL/6J mice were vaccinated via SS or ID injection with MVA-OVA or MVA∆E5R-OVA at a dose of 2 × 10^7^ pfu. One week later, spleens and draining lymph nodes (dLNs) were collected and processed using the Miltenyi GentleMACS™ Dissociator. Splenocytes were stimulated with OVA_257–264_ (SIINFEKL) peptide (5 μg/mL). After 1 h of stimulation, GolgiPlug (BD Biosciences) (1:1000 dilution) was added and incubated for 12 h. Cells were then treated with BD Cytofix/Cytoperm™ kit prior to staining with respective antibodies for flow cytometry analyses. The antibodies used for this assay are as follows: BioLegend: CD3e (145-2C11), CD4 (GK1.5), CD8 (53-5.8), IFN-γ (XMG1.2).

dLNs were digested with collagenase D (2.5 mg/mL) and DNase (50 µg/mL) at 37 °C for 25 min before filtering through a 70-µm cell strainer. For tetramer staining, cells were incubated with tetramers for 30 min at 37 °C. Alexa Fluor 647 H-2K(b) ova 257-264 SIINFEKL tetramer and PE I-A(b) Ova 329-337 AAHAEINEA tetramer were synthesized from NIH Tetramer Core Facility. Cells were analyzed on the BD LSRFortessa.

### Cell lines and primary Cells

BSC40, HEK293T, and MEFs were cultured in Dulbecco’s modified Eagle’s medium (Corning, Cat# 10-014-CV) supplemented with 10% fetal bovine serum (FBS), 2 mM l-glutamine, and 100 U/mL penicillin and 100 μg/mL streptomycin. BHK-21 was cultured in Eagle’s Minimal Essential Medium (Eagle’s MEM, Life Technologies, Cat# 11095-080) containing 10% FBS, with 100 U/mL penicillin and 100 μg/mL streptomycin. For the generation of GM-CSF-BMDCs, bone marrow cells (5 million cells in each 15 cm cell culture dish) were cultured in RPMI-1640 medium supplemented with 10% FBS in the presence of GM-CSF (20 ng/mL, BioLegend, Cat# 576304) for 9-12 days as described previously^43^. For the generation of bone marrow-derived macrophages (BMMs), bone marrow cells were cultured in RPMI-1640 medium supplemented with 10% FBS in the presence of M-CSF (10 ng/mL, PeproTech, Cat# 315-02) for 7–9 days.

Generation of skin dermal fibroblasts: Mice epidermal sheets were removed as previously described^69^. Briefly, skins were washed with cold Ca^2+^ and Mg^2+^ free PBS and then incubated in a digestion buffer containing 1 U dispase/mL at 37 °C for 1 h. Epidermal sheets were mechanically removed, and the remaining dermis was washed in Ca^2+^ and Mg^2+^ free PBS 5 times and incubated in a digestion buffer containing 2 mg/mL collagenase A (Roche), 100 µg/mL of DNase I (Sigma, d4527) and 1% BSA in Ca^2+^ and Mg^2+^ free PBS at 37 °C for 1–2 h. The resulting suspension was filtered through a 100-, 70-, and 40-mm nylon mesh sequentially (VWR) and washed two times with a buffer (Ca^2+^ and Mg^2+^ free PBS containing 1% BSA and 2 mM EDTA). Cells were cultured in RPMI-1640 medium supplemented with 10% FBS. Only the adherent cells were used after 2–3 days of culture.

### Cytokine assays

The IFN-β levels in BALF were determined using a mouse IFN beta ProQuantum Immunoassay kit (ThermoFisher). IFN-α and IFN-β levels in the supernatants of cultured BMDCs were determined by ELISA (PBL Biomedical Laboratories).

### Flow cytometry analysis for DC maturation

GM-CSF-BMDCs from WT or cGAS^−/−^ mice were infected with either MVA-OVA or MVA∆E5R-OVA at an MOI of 10 for 16 h. Cells were washed with MACS buffer (Miltenyi Biotec) and stained with antibodies against CD40 (3/23, Biolegend) and CD86 (GL-1, Biolegend). FACS analyses were performed using LSRFortessa^TM^ Cell Analyzer (BD Biosciences). Data were collected with FACSDiva 8.0 and analyzed with FlowJo software (version 10.5.3).

### Immunofluorescence imaging

Cultured cells were plated in Lab-Tek^TM^ II chamber slide (ThermoFisher) and fixed in 4% (w/v) paraformaldehyde at room temperature (RT) for 10 min, permeabilized with 0.5% (v/v) Triton X-100 in PBS for 5 min, and blocked in 5% goat serum (Sigma), 3% bovine serum albumin (Fisher), and 0.1% Triton X-100 at RT for 1 h. Primary antibodies were incubated at 4 °C at the indicated dilutions overnight: mouse anti-Flag (1:1000, Sigma) and anti-cGAS (1:1000, Abcam). After three washes in PBS, slides were incubated with indicated secondary antibodies, including goat anti-mouse Alexa Fluor-647 (1:1000, Invitrogen) and goat anti-rabbit Alexa Fluor-488 (1:1000, Invitrogen) at RT for 60 min. After three washes in PBS, slides were mounted in ProLong Gold Antifade Mountant (ThermoFisher). Images were acquired using a confocal microscope (Leica TCS SP8 or Zeiss LSM880).

### Live cell imaging

For time-lapse imaging of virisome in MEFs, cells were seeded on Lab-Tek^TM^ II chamber slide (ThermoFisher), infected with MVA∆E5R at MOI 10, and stained with fluorogenic SiR-DNA (Cytoskeleton). Cells were incubated at 37 °C supplemented with 5% CO_2_. Images were acquired using a ZEISS Axio Observer Z1. All the images were further processed with Image J software.

### Plasmid construction

IFN-β reporter plasmid (pIFN-β-luc)^70^ was provided by Michaela Gack (University of Chicago). pRL-TK was purchased from Promega. Human STING expression plasmid was provided by Tom Maniatis (University of Columbia). Murine STING (mSTING) sequences were amplified by PCR and were cloned into pcDNA3.2-DEST plasmids. Human cGAS (hcGAS) and murine cGAS (mcGAS) plasmids were purchased from Invivogen. V5–cGAS were amplified by PCR and were cloned into pcDNA3.2-DEST plasmids. pRK-HA-Ubiquitin-WT, pRK-HA-Ubiquitin-K48, and pBabe 12S E1A were purchased from Addgene. Eighty selected VACV genes were amplified by PCR from the VACV WR genome and subcloned into pcDNA3.2-DEST using the Gateway cloning method (Invitrogen). Flag-tagged E1A and Flag-tagged VACV genes were amplified by PCR and subcloned into pcDNA3.2-DEST using the Gateway cloning method (Invitrogen). VACV E5R (aa: 55-341) was amplified by PCR from the VACV WR genome and subcloned into pcDNA3.1 and pQCXIP.

### Construction of retrovirus expressing vaccinia E5R

HEK293T cells were passaged into a 6-well plate. The next day, cells were transfected with three plasmids–VSVG, gag/pol, and pQCXIP-E5R or pQCXIP with lipofectamine 2000. After 2 days, cell supernatants were collected and filtered through a 0.45 μm filter and stored at −80 °C.

### The Dual-Luciferase reporter assay

The firefly and *Renilla* luciferase activities were measured using the Dual-Luciferase Reporter Assay system according to the manufacturer’s instructions (Promega). To screen for vaccinia inhibitors of the cGAS/STING pathway, cGAS (50 ng) and STING (10 ng) expression plasmids, together with pIFN-β-luc (50 ng), pRL-TK (10 ng), as well as selected vaccinia gene expression plasmid or adenovirus E1A expression plasmid (200 ng) were transfected into HEK293T cells. Twenty-four hours post-transfection, cells were collected and lysed. To assess the effects of vaccinia inhibitors of the STING pathway, STING (50 ng) expression plasmid, together with pIFN-β-luc (50 ng), pRL-TK (10 ng), as well as selected vaccinia gene expression, constructs or adenovirus E1A expression plasmid (200 ng) were transfected into HEK293T cells. The relative luciferase activity was expressed as arbitrary units by normalizing firefly luciferase activity under the IFNB promoter to *Renilla* luciferase activity from a control plasmid, pRL-TK.

### Western blot analysis

Cells were lysed in RIPA lysis buffer supplemented with 1× Halt™ Protease and Phosphatase Inhibitor Cocktail (ThermoFisher). To extract cytoplasmic and nuclear proteins, cells were processed with the NE-PER Nuclear and Cytoplasmic Extraction Kit (ThermoFisher, 78833). Protein samples were separated by SDS-PAGE and then transferred to nitrocellulose membrane and incubated with primary antibodies specific for cGAS (CST, 31659), STING (CST, 13647), FLAG (Sigma, F3165), HA (Sigma, H3663), V5 (ThermoFisher, R960-25), mCherry (ThermoFisher, M11217), β-actin (Sigma, A2228), Lamin B1 (Sigma, ZRB1143) and GAPDH (CST, 2118) were used. C7 antibody was produced in our lab and described before^24^. E3 antibody was kindly provided by Dr. Stuart N. Isaacs (University of Pennsylvania)^71^. HRP-conjugated anti-rabbit, mouse, or rat IgG antibodies were used as secondary antibodies (CST, 7074, 7076, or 7077). Detection was performed using SuperSignal^TM^ Substrates (Thermo Fisher, 34577 or 34095).

### Detection of ubiquitinated cGAS proteins

To detect ubiquitinated cGAS protein during vaccinia virus infection, BMDCs were infected with MVA at MOI 10 for 6 h in the presence of MG132 (25 μg/mL). Ubiquitinated proteins were purified using Halo-4× UBA^UBQLN1^ as previously described^40^. Briefly, whole-cell extracts (1 mg) were lysed in lysis buffer containing 100 mM chloroacetamide and incubated at 4 °C for 16 h with 30 μL of Halo-4× UBA^UBQLN1^ beads (pack volume). Following four washes with lysis buffer containing 1 M NaCl, and one final wash in 10 mM Tris (pH 8.0), proteins were released from Halo-4xUBA^UBQLN1^ using sample buffer prior to analysis by SDS-PAGE. For cGAS and E5R co-transfection, HEK293T cells in 10-cm plates were transfected with V5–cGAS together with E5R-Flag or pcDNA3.1. 24 h later, cells were treated with MG132 (25 μg/mL) for 6 h before lysing in lysis buffer.

### Co-immunoprecipitation

For cGAS ubiquitination assays, HEK293T cells in 10-cm plates were transfected with V5–cGAS together with HA-Ub-WT or HA-Ub-K48. 24 h later, cells were infected with MVA or MVA∆E5R at MOI 10 for 6 h in the presence of MG132 (25 μg/mL) and lysed in RIPA lysis buffer (ThermoFisher, 89901) on ice for 30 min. Anti-V5 antibody (ThermoFisher, R960-25) was added into cell lysate to a final concentration of 1 μg/mL and incubated at 4 °C overnight on a rotator. The next day, protein G-magnetic beads (Bio-Rad, 161-4023) were added and incubated at 4 °C for 2 h. The beads were washed five times with RIPA buffer. Lastly, the bead-bound proteins were denatured in SDS buffer by heating at 98 °C for 5 min before loading on an SDS-PAGE gel.

For cGAS and E5 interaction assay, HEK293T cells in 10 cm plates were transfected with V5–cGAS together with E5R-Flag or pcDNA3.1. Two days later, cells were lysed in Pierce IP lysis buffer on ice for 30 min. Cell lysates were treated with or without 250 U/mL Benzonase (Sigma, E8263) at 4 °C for 30 min before being mixed with protein G-agarose (ThermoFisher, 20399). In BMDCs, cells were infected with MVA∆E5R-E5R-Flag at an MOI of 10 for 6 h. Cells were lysed as above. cGAS antibody (Abcam, ab252416) was added into cell lysate to a final concentration of 1 μg/ml and incubated at 4 °C overnight on a rotator. The next day, protein G-agarose beads were added and incubated at 4 °C for 2 h. The beads were washed five times with IP lysis buffer. Lastly, proteins in the SDS buffer were denatured by heating at 98 °C for 5 min before they were loaded on SDS-PAGE.

### Quantitative real-time PCR

Total RNA was extracted from whole cell lysates using TRIzol reagent (Invitrogen) or with RNeasy Plus Mini kit (Qiagen). RNAs were reverse-transcribed and amplified by PCR using the Verso cDNA synthesis kit (Thermo Fisher) and SYBR^TM^ Green Master Mix (Thermo Fisher). Cellular RNAs were normalized to GAPDH levels. GAPDH levels were stable in BMDCs under virus infection^43^. The ΔΔCt method was used to measure the expression of genes, and the efficiencies of qPCR assays were between 90% and 110%^72^. All assays were performed on an ABI 7500 system and analyzed with ABI 7500 SDS software v.1.3 (Applied Biosystems) and followed MIQE^73^. Data distribution was assessed with the Shapiro–Wilk test. Primer sequences are listed in Table S1.

### cGAMP measurement by liquid chromatography-mass spectrometry (LC-MS)

cGAMP was measured by LC–MS as previously reported^74^. Briefly, cell pellets were supplemented with 80 fmol internal standards (^15^N_10_-cGAMP, in-house generated) and were subsequently extracted in 80% methanol and 2% acetic acid and twice in 2% acetic acid to obtain metabolite extract. cGAMP was enriched from combined extracts on HyperSep Aminopropyl SPE Columns (Thermo Scientific). After washing twice in 2% acetic acid and once in 80% methanol, samples were eluted in 4% ammonium hydroxide in 80% methanol. Vacuum-dried eluents were dissolved in water and analyzed on a Dionex U3000 HPLC coupled with a TSQ Quantiva Triple Quadruple mass spectrometer (Thermo Scientific). The chromatography used LUNA NH_2_ resin (5 µm, Phenomenex) as stationary phase packed in 0.1 mm ID × 70 mm L silica capillaries. Mobile phases are acetonitrile (A), 20 mM ammonium bicarbonate, and 20 mM ammonium hydroxide aqueous solution (B). Flow rate is 800 nL/min (0–4 min), 300 nL/min (4–19 min), and 600 nL/min (19–27 min), with a gradient of 20% B (0–3 min), 50% B (4 min), 80% B (14–18 min), and 20% B (19–27 min). cGAMP and standard were analyzed by multiple reaction monitoring in the positive mode with the following transitions: 675-136, 675-152, 675-476, and 675-524 for cGAMP; and 685-136, 685-157, 685-480, and 685-529 for the ^15^N_10_-cGAMP standard. Endogenous cGAMP levels were calculated by multiplying the cGAMP-to-standard ratios by 80 fmol (the amount of standard spiked into each sample).

### RNA-seq analyses of GM-CSF-cultured BMDCs infected with MVA vs. MVA∆E5R

GM-CSF-cultured BMDCs (1 × 10^6^) from WT or cGAS^−/−^ mice were infected with MVA or MVA∆E5R at a multiplicity of infection (MOI) of 10. Cells were collected at 16 h post-infection. Total RNA was extracted from collected cells at indicated time points using RNeasy Plus Mini Kit (Qiagen) according to the manufacturer’s protocol, including DNase I treatment. Total RNA integrity was analyzed using a 2100 Bioanalyzer (Agilent Technologies). Messenger RNA was prepared using TruSeq Stranded mRNA Sample Library Preparation kit (Illumina, San Diego, CA) according to the manufacturer’s instructions. The normalized final cDNA libraries were pooled and sequenced on Illumina NovaSeq6000 sequencer with pair-end 50 cycles. The raw sequencing reads in BCL format was processed through bcl2fastq 2.19 (Illumina) for FASTQ conversion and demultiplexing. After trimming the adaptors with cutadapt (version 1.18), RNA reads was aligned and mapped to the GRCh38 human reference genome by STAR (Version 2.5.2), and transcriptome reconstruction was performed by Cufflinks (Version 2.1.1). The abundance of transcripts was measured with Cufflinks in Fragments Per Kilobase of the exon model per Million mapped reads (FPKM). Gene expression profiles were constructed for differential expression, cluster, and principle component analyses with the DESeq2 package. GSEA software from the Broad Institute was used to identify functions of differentially expressed genes. Genes were ranked by the log2 FC value obtained from differential expression analysis, and the preranked version of the tool was used to identify significantly enriched biological pathways. Heatmaps of significantly enriched biological pathways were generated with the R package pheatmap (https://www.rdocumentation.org/packages/pheatmap/versions/1.0.12/topics/pheatmap).

Volcano plots were generated with the R package EnhancedVolcano (https://bioconductor.org/packages/release/bioc/html/EnhancedVolcano.html).

The RNA-Seq data for this project have been deposited in NCBI’s Gene Expression Omnibus, accession number GSE185431.

### Statistics

Two-tailed unpaired Student’s *t*-test was used for comparisons of two groups in the studies. Survival data were analyzed by log-rank (Mantel-Cox) test. The *p* values deemed significant are indicated in the figures as follows: *p < 0.05; **p < 0.01; ***p < 0.001; ****p < 0.0001. The numbers of animals included in the study are discussed in each figure legend.

### Biological materials

All unique materials are readily available from the corresponding authors upon request. The availability of the antibody recognizing VACV protein C7 is limited.

### Reporting summary

Further information on research design is available in the Nature Portfolio Reporting Summary linked to this article.

## Supplementary information

Supplementary Information

Reporting Summary

## Data Availability

RNAseq data have been deposited in the Gene Expression Omnibus (GEO) under the accession number GSE185431. All the other data supporting the findings of this study are available within the article and its supplementary information files. Source data are provided in this paper.

## Source data

Source Data
