# Supplementary material for: Vaccinia E5 is a major inhibitor of the DNA sensor cGAS
Source: Nat Commun. 2023 May 22;14:2898. doi: 10.1038/s41467-023-38514-5 (PMC10201048; doi:10.1038/s41467-023-38514-5)
Supplement: Supplementary file 1 — Supplementary Information [file 41467_2023_38514_MOESM1_ESM.pdf]

Supplementary Information

Vaccinia E5 is a major inhibitor of the DNA sensor cGAS

Ning Yang[1*], Yi Wang[1], Peihong Dai[1], Tuo Li[2], Christian Zierhut[4,5], Adrian Tan[6], Tuo Zhang[6], Jenny Zhaoying Xiang[6], Alban Ordureau[7], Hironori Funabiki[4], Zhijian Chen[3], and Liang Deng[1,2,8*#]

[1]Dermatology Service, Department of Medicine, Memorial Sloan Kettering Cancer Center, New York, NY 10065, USA

[2]Immuno-oncology service, Human Oncology and Pathogenesis Program; Memorial Sloan Kettering Cancer Center, New York, NY 10065, USA

[3]Department of Molecular Biology, University of Texas Southwestern Medical Center, Dallas, TX, 75390, USA

[4]Laboratory of Chromosome and Cell Biology, The Rockefeller University, New York, NY 10065, USA

[5]Present address: The Institute of Cancer Research, London, SW3 6JB, UK

[6]Genomic Resources Core Facility, Weill Cornell Medical College, New York, NY, 10065, USA

[7]Cell Biology Program, Sloan Kettering Institute, Memorial Sloan Kettering Cancer Center, New York, NY 10065, USA

[8]Weill Cornell Medical College, New York, NY 10065, USA

*corresponding authors. [#]Lead contact. Mailing address for Liang Deng and Ning Yang: Dermatology Service, Department of Medicine, Memorial Sloan Kettering Cancer Center, 1275 York Ave., New York, NY 10065. Email: dengl@mskcc.org; yangn@mskcc.org.

This file contains:

- Supplementary Fig. 1
- Supplementary Fig. 2
- Supplementary Fig. 3
- Supplementary Fig. 4
- Supplementary Fig. 5

- Supplementary Fig. 6
- Supplementary Fig. 7
- Supplementary Table 1
- Supplementary Table 2

Supplementary Fig. 1

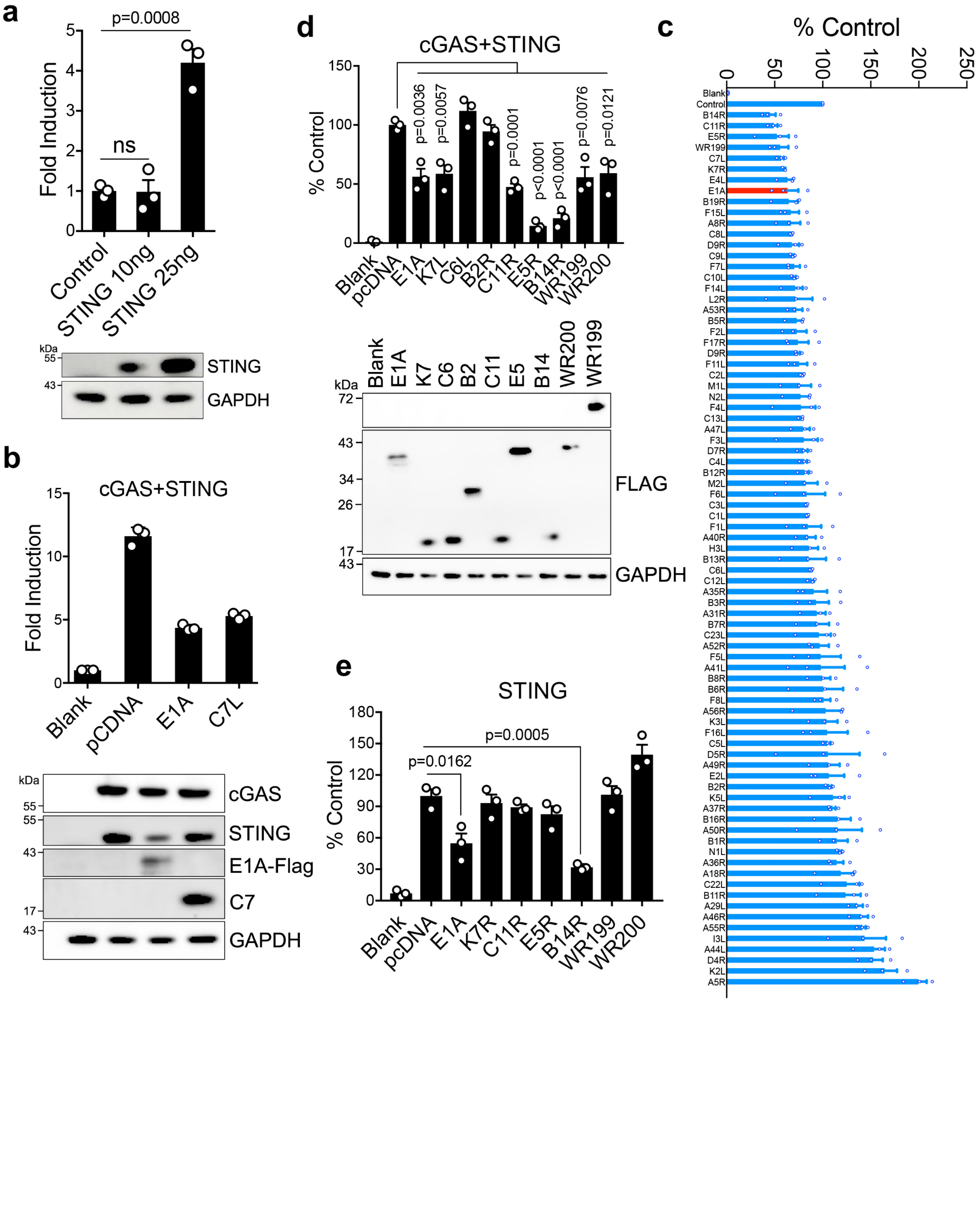

Supplementary Fig. 1 A dual-luciferase assay to screen for vaccinia viral inhibitors of the cGAS-STING-mediated type I IFN pathway. **a,** Dual-luciferase assay of HEK293T cells transfected with Ifnb1-firefly luciferase reporter, pRL-TK expressing Renilla luciferase, and STING-expressing plasmids. Cells were harvested at 24 h post-transfection, and luminescence was determined and expressed as % control. Cell lysates were analyzed by immunoblot. n=3 independent samples. **b,** Dual-luciferase assay of HEK293T cells transfected with Ifnb1-firefly luciferase reporter, pRL-TK expressing Renilla luciferase, cGAS (50 ng) and STING (10 ng)-expressing plasmids, Adenovirus E1A-expressing, vaccinia C7L expressing or pcDNA3.1 control plasmid. Cells were harvested at 24 h post-transfection, and luminescence was determined and expressed as % control. Cell lysates were analyzed by immunoblot. n=3 independent samples. **c,** A dual-luciferase assay to screen for vaccinia viral inhibitors of the cGAS-STING-mediated type I IFN pathway. HEK293T cells were transfected with an IFNB-firefly luciferase reporter, a control plasmid pRL-TK expressing *Renilla* luciferase, cGAS (50 ng) and STING (10 ng)-expressing plasmids, individual vaccinia protein-expressing plasmid (200 ng), or pcDNA3.1 control plasmid. Adenovirus E1A-expressing plasmid was used as a positive control. Cells were harvested at 24 h post-transfection, and luminescence was determined and expressed as % control. n=3 independent samples. **d,** Same as C. Flag-tagged vaccinia protein-expressing plasmids were used for transfection. A dual-luciferase assay to verify potential vaccinia viral inhibitors of the cGAS-STING pathway. Cell lysates were analyzed by immunoblot. n=3 independent samples. **e,** A dual-luciferase assay to verify potential vaccinia viral inhibitors of the STING-IFNB pathway. STING-expressing plasmid (50 ng) was co-transfected with an IFNB-firefly luciferase reporter, a control plasmid pRL-TK expressing *Renilla* luciferase. n=3 independent samples. Two-tailed unpaired Student's *t* test was used for comparions of two groups in the studies. Data are presented as mean ± SEM. Source data are provided as a Source Data file.

Supplementary Fig. 2

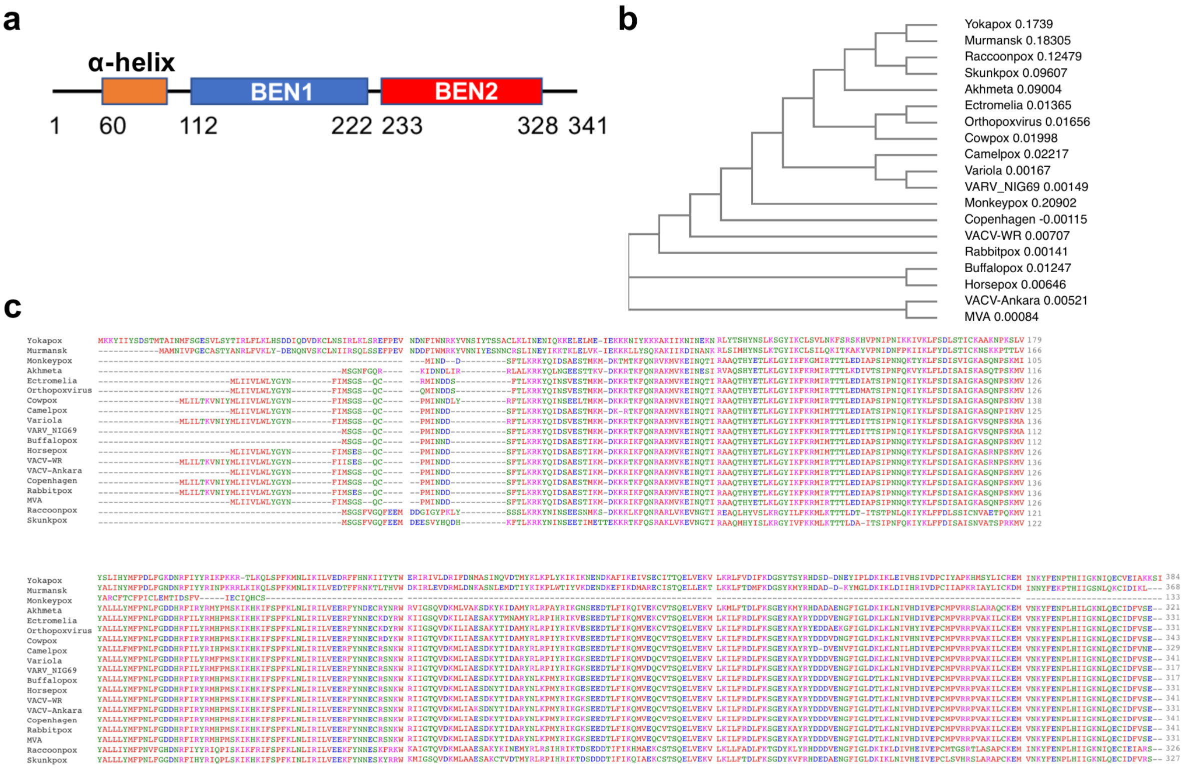

Supplementary Fig. 2 Sequence alignment of E5 from orthopoxviruses. a, Schematic diagram of E5 protein, which is comprised of an N-terminal α-helical and two C-terminal BEN domains. **b**, Phylogenetic tree of E5 sequence alignments. **c**, Multiple sequence alignments of E5 from orthopoxviruses.

Supplementary Fig. 3

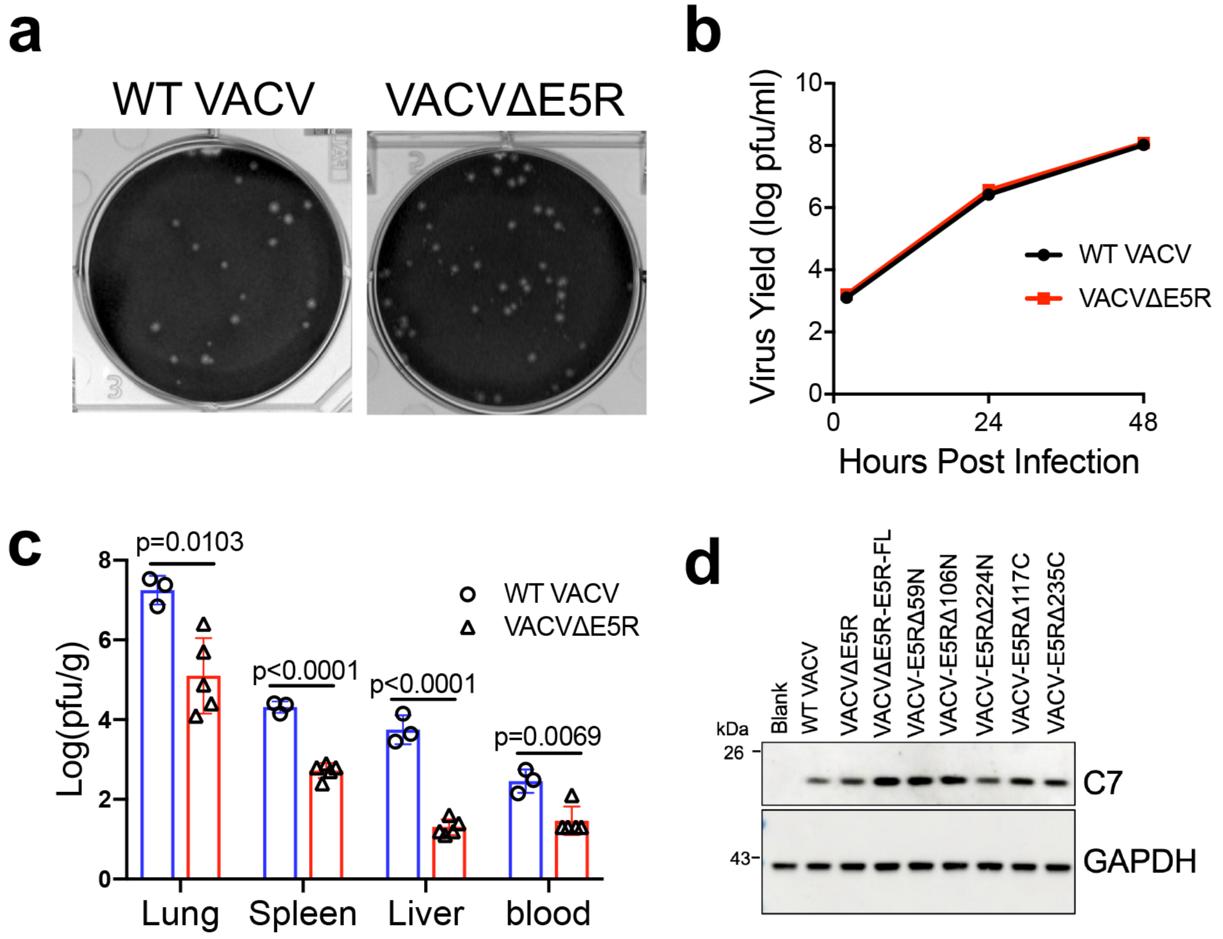

Supplementary Fig. 3 Viral replication of WT VACV and VACVΔE5R in vitro.

a, Viral plaques of WT VACV and VACVΔE5R at day 2 post infection in BSC40 cells. **b**, Viral growth curve in BSC40 cells at an initial MOI of 0.05. **c**, Titers of WT VACV or VACVΔE5R in the lungs, livers, spleens and blood of WT C57BL/6J mice at day 6 post intranasal infection with WT VACV at a dose of 2 x 10^5 pfu or VACVΔE5R at a dose of 2 x 10^7 pfu**.** n=3-5 independent samples. **d**, Immunoblot of C7 in BMDCs from WT mice infected with different vaccinia viruses at MOI of 10 for 6 h. Two-tailed unpaired Student's *t* test was used for comparions of two groups in the studies. Data are presented as mean ± SEM. Source data are provided as a Source Data file.

Supplementary Fig. 4

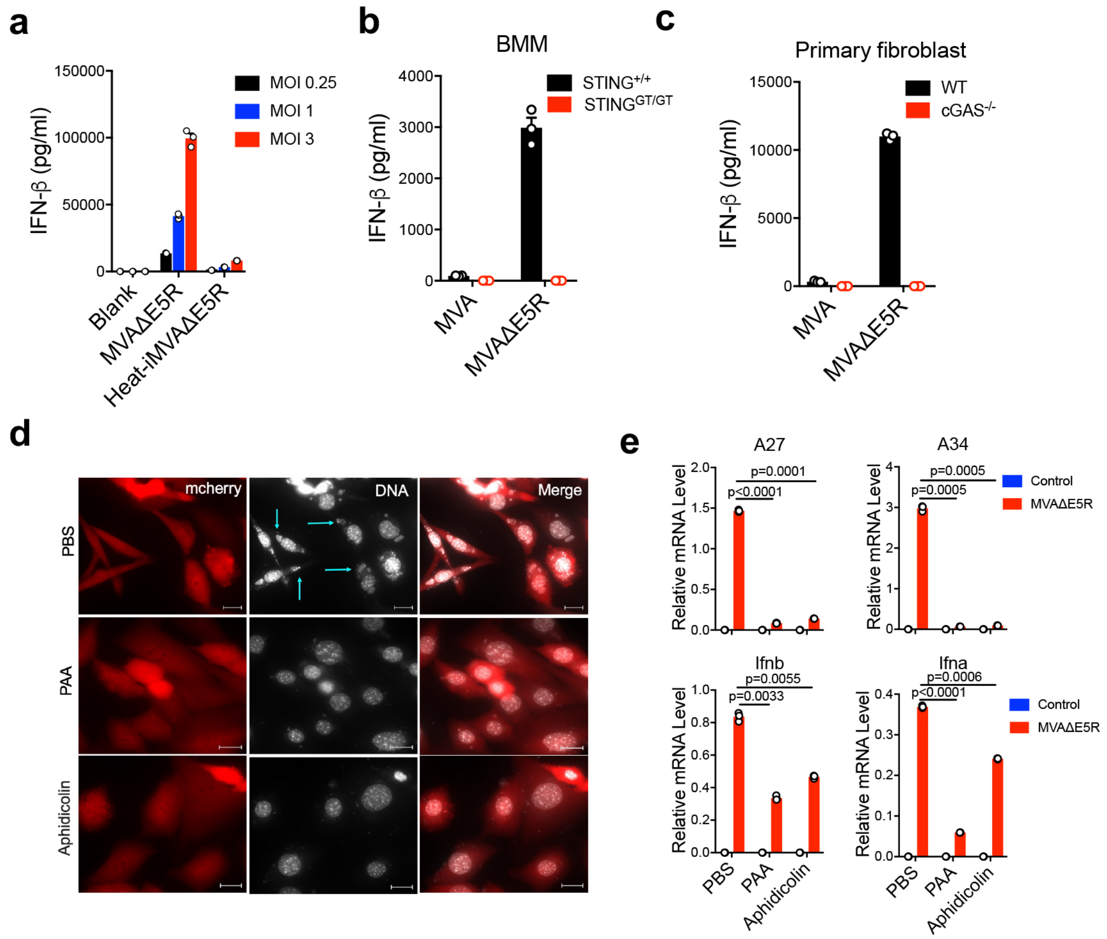

Supplementary Fig. 4 MVAΔE5R strongly induces type I IFN production in a cGAS/STING-dependent manner. **a**, ELISA analyses of IFN-β levels in the supernatants of WT BMDC infected with MVAΔE5R or Heat-iMVAΔE5R at different MOI for 16 h. n=3 independent samples. **b**,**c**, ELISA analyses of IFN-β levels in the supernatants of WT or STING$^{Gt/Gt}$ BMM (**b**) or WT or cGAS$^{-/-}$ primary fibroblast (**c**) infected with MVA, or MVAΔE5R at a MOI of 10 for 16 h. n=3 independent samples. **d**, Representative images showing virosome in MEF cells treated with PAA, Aphidicolin or PBS after infection with MVAΔE5R at a MOI of 10 for 6 h. Scale bar, 15 µm. **e**, RT-PCR of *Ifnb*, *Ifna*, A27, and A34 gene expression of BMDC from WT mice infected with MVAΔE5R or PBS control at a MOI of 10 for 6 h. The cells were treated with PAA, Aphidicolin or PBS during virus infection. n=3 independent samples. Two-tailed unpaired Student's *t* test was used for comparions of two groups in the studies. Data are presented as mean ± SEM. Source data are provided as a Source Data file.

Supplementary Fig. 5

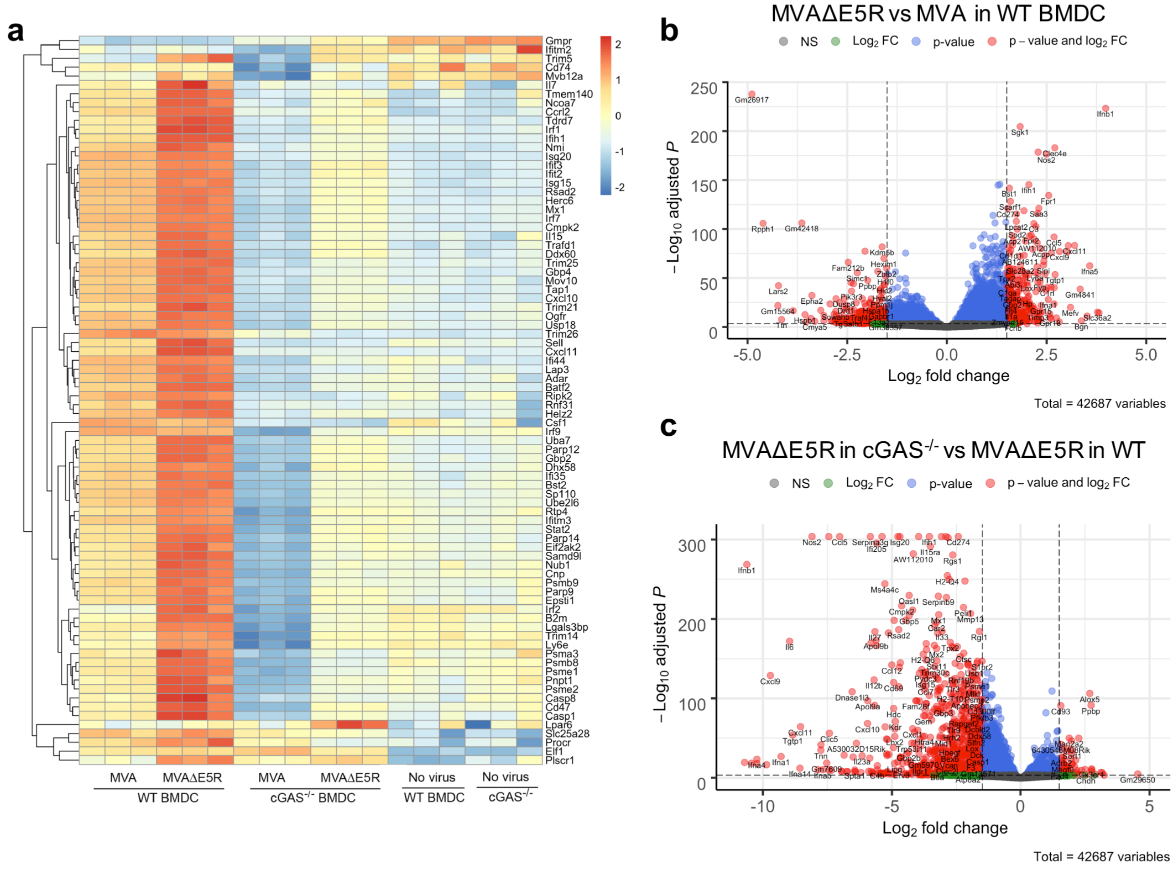

Supplementary Fig. 5 MVAΔE5R-OVA promotes DC maturation and antigen-specific CD8^{+} T cells activation. a, A heatmap showing the expression of genes belong to the hallmark of Interferon Alpha response gene set based on differentially expressed genes (DEGs) in WT or *cGas*$^{-/-}$ BMDCs infection with MVA vs. MVAΔE5R. **b**, A volcano plot showing DEGs in WT BMDCs infected with MVAΔE5R vs. MVA. **c**, A volcano plot showing DEGs in MVAΔE5R-infected WT vs. *cGas*$^{-/-}$ BMDCs. Two-tailed unpaired multiple *t* test was used for comparions of two groups in the studies.

Supplementary Fig. 6.

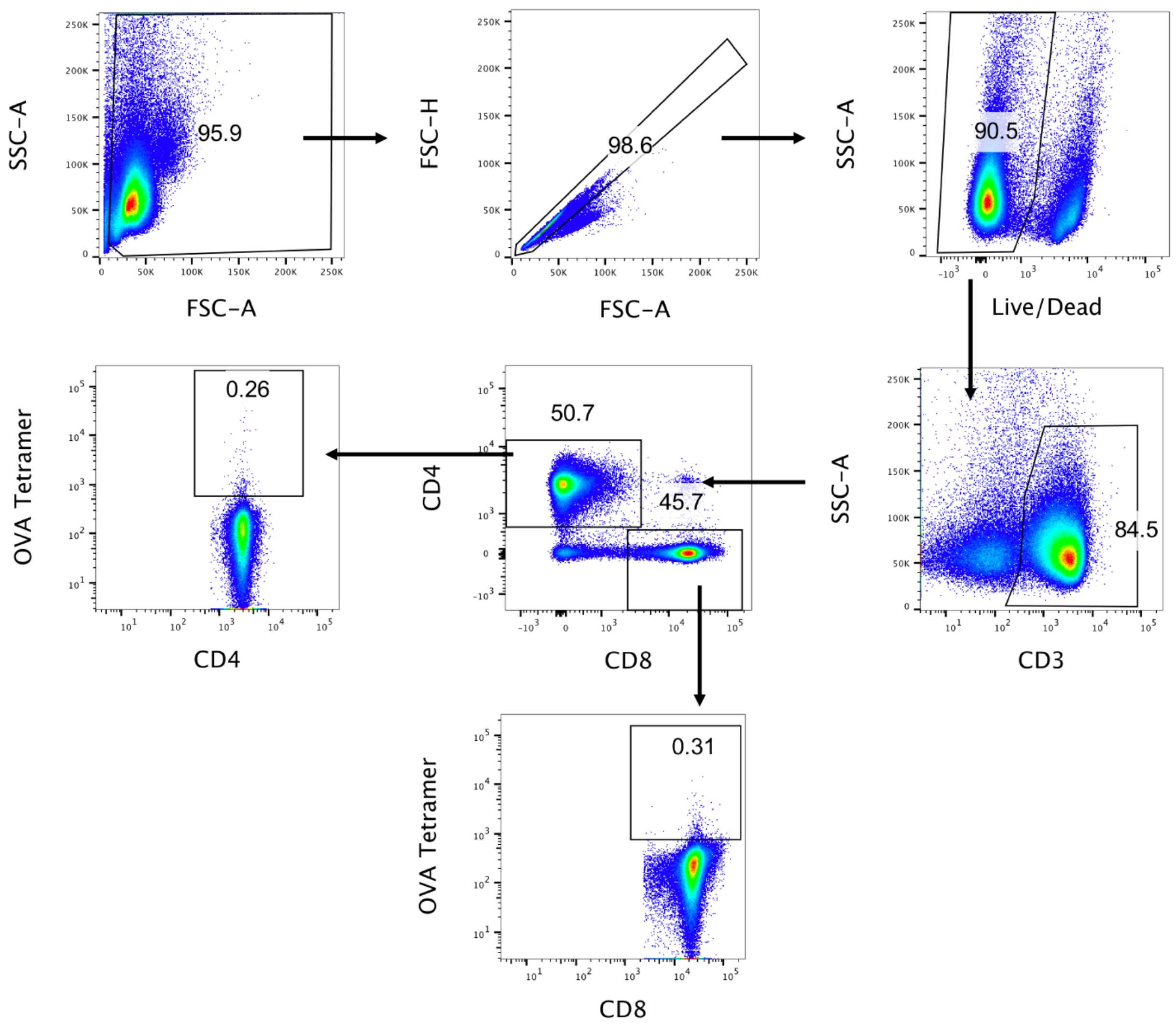

Supplementary Fig. 6 Gating strategy to define CD8^{+} and CD4^{+} T cell populations in the spleen and lymph nodes. Within the single cell suspension, doublets and dead cells were excluded from analysis. CD3^{+} CD4^{+} represents CD4^{+} T cells. CD3^{+} CD8^{+} represents CD8^{+} T cells.

Supplementary Fig. 7.

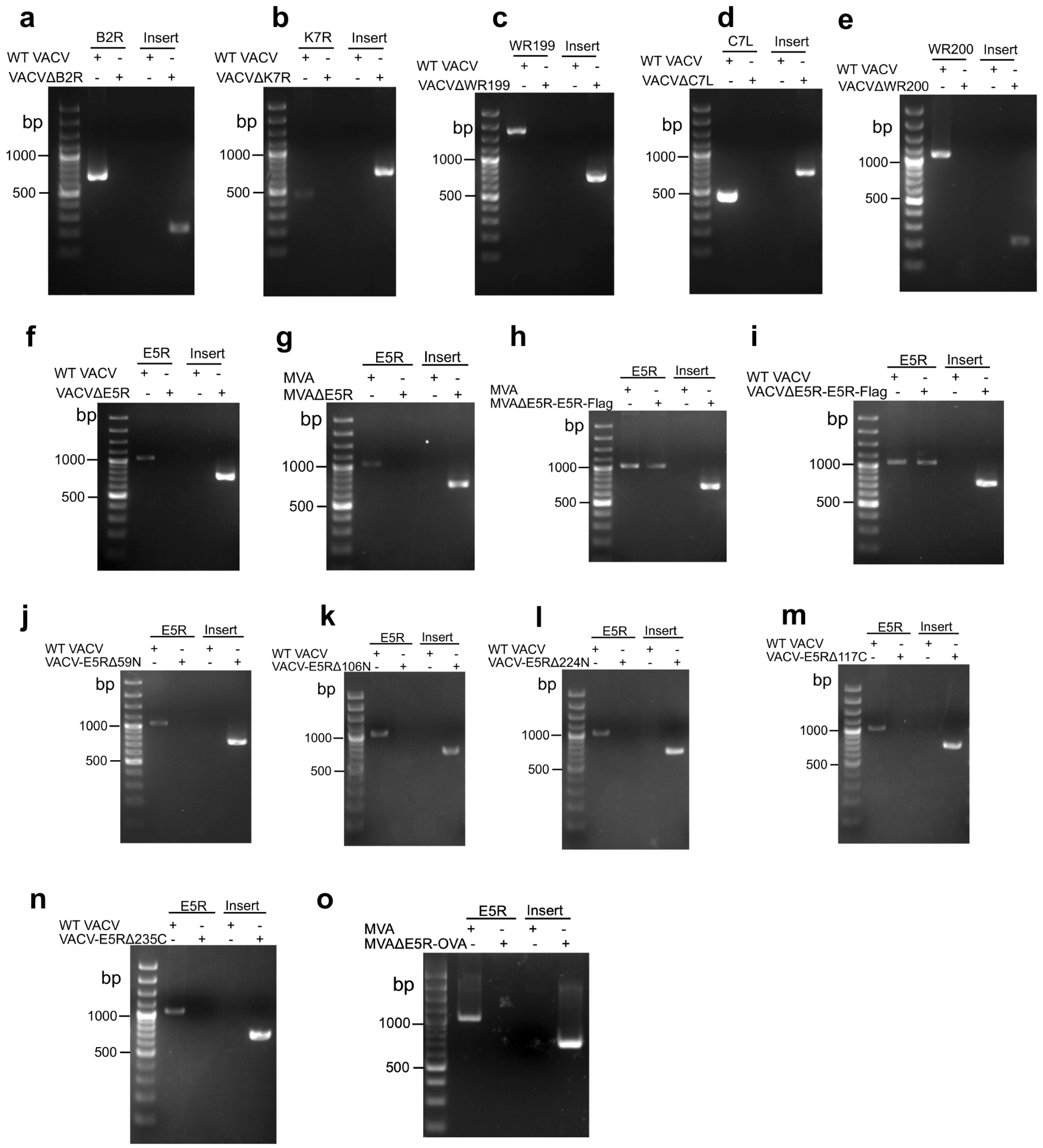

Supplementary Fig. 7 PCR analysis of purified recombinant vaccinia viruses.

PCR of viral genomic DNA to amplify viral genes and recommbinant plasmid fragment. **a**, B2R and Insert (GFP). **b**, K7R and insert (mcherry). **c**, WR199 and Insert (mcherry). **d**, C7L and Insert (mcherry). **e**, WR200 and Insert (GFP). **f-o**, E5R and Insert (mcherry). Data are representative of two **a-o** independent experiments.

Supplementary Table 1

List of primers used in this paper for PCR.

Primer sequence	SOURCE
mcherry For: ATGGTGAGCAAGGGCGAGGAGG	Integrated DNA technologies IDT
mcherry Rev: ACAATTCGTGATCAGCTCGTCC	
GFP For: ATGGAGAGCGACGAGAGCGG	
GFP Rev: GCGTGCAGGAAGGGGTTCTC	
B2R For: ATGGCGATGTTTTACGCACACGC	
B2R Rev: TTACCTCGATGGTGCCGCCGATATAG	
E3L For: ATGTCTAAAATCTATATCGACGAG	
E3L Rev: TCAGAATCTAATGATGACGTAACC	
WR199 For: ATGAGTCGTCGTCTGATTTATG	
WR199 Rev: CTATACTTTGGTAGGTGGATACG	
WR200 For: ATGACGATGAAAATGATGGTAC	
WR200 Rev: TTACTCCAATACTACTGTAGTTG	
K7R For: ATGGCGACTAAATTAGATTATGAGG	
K7R Rev: TCAATTCAATTTTTTTTCTAG	
C7L For: ATGGGTATACAGCACGAATTC	
C7L Rev: TTAATCCATGGACTCATAATCTC	
E5R For: ATGTTGATATTAACAAAAGTG	
E5R Rev: CTATTCACTAACAAAGTCAATGC	

Supplementary Table 2

List of primers used in this paper for qRT-PCR.

**Primer sequence**	SOURCE
qPCR *Ifnb* For: TGGAGATGACGGAGAAGATG	Integrated DNA technologies IDT
qPCR *Ifnb* Rev: TTGGATGGCAAAGGCAGT	
qPCR *Ccl4* For: GCCCTCTCTCTCCTCTTGCT	
qPCR *Ccl4* Rev: CTGGTCTCATAGTAATCCATC	
qPCR *Gapdh* For: AGGTCGGTGTGAACGGATTTG	
qPCR *Gapdh* Rev: TGTAGACCATGTAGTTGAGGTCA	
qPCR *Ccl5* For: GCCCACGTCAAGGAGTATTTCTA	
qPCR *Ccl5* Rev: ACACACTTGGCGGTTCCTTC	
qPCR *Ifna* For: TCTGATGCAGCAGGTGGG	
qPCR *Ifna* Rev: AGGGCTCTCCAGACTTCTGCTCTG	
qPCR *A34* For: GGCATAGGAACATTTCTGCATTAC	
qPCR *A34* Rev: TACGACACTGATAAACCGCATT	
qPCR *A27* For: CCGTCCAGTCTGAACATCAAT	
qPCR *A27* Rev: GTGTTGTAAACGCAACGATGAA	
qPCR *E3* For: GCAGAGATTGTGTGTGCGGCTATT	
qPCR *E3* Rev: GGTGACAGGGTTAGCATCTTTCCA	
