# Supplementary material for: Vaccinia E5 is a major inhibitor of the DNA sensor cGAS
Source: Nat Commun. 2023 May 22;14:2898. doi: 10.1038/s41467-023-38514-5 (PMC10201048; doi:10.1038/s41467-023-38514-5)
Supplement: Supplementary file 2 — Reporting Summary [file 41467_2023_38514_MOESM2_ESM.pdf]

nature portfolio

Corresponding author(s): NCOMMS-22-21911C

Last updated by author(s): May 1, 2023

Reporting Summary

Nature Portfolio wishes to improve the reproducibility of the work that we publish. This form provides structure for consistency and transparency in reporting. For further information on Nature Portfolio policies, see our Editorial Policies and the Editorial Policy Checklist.

Statistics

For all statistical analyses, confirm that the following items are present in the figure legend, table legend, main text, or Methods section.

n/a	Confirmed	
☐	☒	The exact sample size (n) for each experimental group/condition, given as a discrete number and unit of measurement
☐	☒	A statement on whether measurements were taken from distinct samples or whether the same sample was measured repeatedly
☐	☒	The statistical test(s) used AND whether they are one- or two-sided<br>*Only common tests should be described solely by name; describe more complex techniques in the Methods section.*
☒	☐	A description of all covariates tested
☐	☒	A description of any assumptions or corrections, such as tests of normality and adjustment for multiple comparisons
☐	☒	A full description of the statistical parameters including central tendency (e.g. means) or other basic estimates (e.g. regression coefficient) AND variation (e.g. standard deviation) or associated estimates of uncertainty (e.g. confidence intervals)
☐	☒	For null hypothesis testing, the test statistic (e.g. F, t, r) with confidence intervals, effect sizes, degrees of freedom and P value noted<br>*Give P values as exact values whenever suitable.*
☒	☐	For Bayesian analysis, information on the choice of priors and Markov chain Monte Carlo settings
☒	☐	For hierarchical and complex designs, identification of the appropriate level for tests and full reporting of outcomes
☒	☐	Estimates of effect sizes (e.g. Cohen's d, Pearson's r), indicating how they were calculated

Our web collection on statistics for biologists contains articles on many of the points above.

Software and code

Policy information about availability of computer code

Data collection	FACSDiva 8.0, ABI 7500 SDS software v.1.3
Data analysis	Flowjo 10.5.3 (Tree Star) for FACS results; GraphPad Prism 7 for statistics; Leica TCS SP8 and Image J (1.53) for imaging data analysis

For manuscripts utilizing custom algorithms or software that are central to the research but not yet described in published literature, software must be made available to editors and reviewers. We strongly encourage code deposition in a community repository (e.g. GitHub). See the Nature Portfolio guidelines for submitting code & software for further information.

Data

Policy information about availability of data

All manuscripts must include a data availability statement. This statement should provide the following information, where applicable:

- Accession codes, unique identifiers, or web links for publicly available datasets
- A description of any restrictions on data availability
- For clinical datasets or third party data, please ensure that the statement adheres to our policy

All data generated and supporting the findings of this study are available within this paper. RNA-seq data has been deposited in the NCBI Gene Expression Omnibus (GEO) database and are accessible through the GEO accession number: GSE185431. All the other data supporting the findings of this study is available within the article and its supplementary information files. Source data are provided with this paper.

Research involving human participants, their data, or biological material

Policy information about studies with human participants or human data. See also policy information about sex, gender (identity/presentation), and sexual orientation and race, ethnicity and racism.

Reporting on sex and gender	N/A
Reporting on race, ethnicity, or other socially relevant groupings	N/A
Population characteristics	N/A
Recruitment	N/A
Ethics oversight	N/A

Note that full information on the approval of the study protocol must also be provided in the manuscript.

Field-specific reporting

Please select the one below that is the best fit for your research. If you are not sure, read the appropriate sections before making your selection.

☒ Life sciences ☐ Behavioural & social sciences ☐ Ecological, evolutionary & environmental sciences

For a reference copy of the document with all sections, see nature.com/documents/nr-reporting-summary-flat.pdf

Life sciences study design

All studies must disclose on these points even when the disclosure is negative.

Sample size	Sample sizes were not predetermined and are indicated in the figure legends. The group sizes of mice and samples were chosen based on our experience with similar studies, common practice in this field and resource availability. Reference: Yang N et al. "Lung type II alveolar epithelial cells collaborate with CCR2+ inflammatory monocytes in host defense against poxvirus infection." Nature Communications 13.1 (2022): 1671.
Data exclusions	We did not exclude data in this study.
Replication	All the experimental findings were reliably reproduced as validated by at least two independent experiments.
Randomization	Samples were randomized into experimental or control groups. Animals were randomized into different treatment groups.
Blinding	Investigators were not blinded to group allocation during data collection and/or analysis in other experiments, because the same researcher performed the experiment and analyzed the data.

Reporting for specific materials, systems and methods

We require information from authors about some types of materials, experimental systems and methods used in many studies. Here, indicate whether each material, system or method listed is relevant to your study. If you are not sure if a list item applies to your research, read the appropriate section before selecting a response.

Materials & experimental systems

n/a	Involved in the study	
☐	☒	Antibodies
☐	☒	Eukaryotic cell lines
☒	☐	Palaeontology and archaeology
☐	☒	Animals and other organisms
☒	☐	Clinical data
☒	☐	Dual use research of concern
☒	☐	Plants

Methods

n/a	Involved in the study	
☒	☐	ChIP-seq
☐	☒	Flow cytometry
☒	☐	MRI-based neuroimaging

Antibodies

Antibodies used	The following antibodies were used for flow cytometry: BioLegend: CD45.2 (104) Cat# 109822, CD3e (145-2C11) Cat# 100341, CD4

Antibodies used	(GK1.5) Cat# 100428, CD8 (53-5.8) Cat# 140418, IFN-r (XMG1.2) Cat# 505810, CD86(GL-1) Cat# 105030, CD40 (3/32) Cat# 124610. Thermo Fisher: CD16/CD32 (93) Cat# 14-0161-82. All antibodies are used at 1:200.<br>The following antibodies were used for imaging: anti-GFP (Abcam ab13970), anti-Flag (Sigma F3165), anti-mcherry (ThermoFisher M11217), anti-cGAS (Abcam ab252416). Goat anti-Chicken IgY (H+L) Secondary Antibody, Alexa Fluor 488 (ThermoFisher A-11039), Goat anti-Rat IgG (H+L) Cross-Adsorbed Secondary Antibody, Alexa Fluor 594 (ThermoFisher A-11007), Goat anti-Mouse IgG (H+L) Highly Cross-Adsorbed Secondary Antibody, Alexa Fluor 488 (ThermoFisher A-28175), Goat anti-Mouse IgG (H+L) Highly Cross-Adsorbed Secondary Antibody, Alexa Fluor 647 (ThermoFisher A-21235). All antibodies are used at 1:1000 unless otherwise noted.<br>The following antibodies were used for western blot: anti-FLAG (Sigma, F3165, clone: M2, 1:1000), anti-GAPDH (CST, 2118, clone: 14C10, 1:2000), anti-Lamin B1 (Sigma, ZRB1143, clone: 6K22, 1:1000), anti-cGAS (CST, 31659, clone: D3O8O, 1:1000), anti-anti-STING (CST, 13647 clone: D2P2F 1:1000), anti-HA (Sigma, H3663, clone: HA-7, 1:1000), anti-V5 (ThermoFisher, R960-25, 1:1000), anti-b-actin (Sigma, A2228, clone: AC-74, 1:2000) and Anti-rabbit, mouse or rat HRP-linked IgG antibody (CST 7074, 7076 or 7077, 1:5000).
Validation	The specificities of listed FACS antibodies have been validated by the manufacturer by flow cytometry.<br>CD45.2 (104) Cat# 109822: https://www.biolegend.com/en-us/products/alexa-fluor-700-anti-mouse-cd45-2-antibody-3393?GroupID=BLG1934<br>CD3e (145-2C11) Cat# 100341: https://www.biolegend.com/en-us/products/brilliant-violet-421-anti-mouse-cd3epsilon-antibody-7132?GroupID=BLG6744<br>CD4 (GK1.5) Cat# 100428: https://www.biolegend.com/en-us/products/pacific-blue-anti-mouse-cd4-antibody-3316?GroupID=BLG4745<br>CD8 (53-5.8) Cat# 140418: https://www.biolegend.com/en-us/products/percp-cyanine5-5-anti-mouse-cd8b2-antibody-17484?GroupID=BLG8876<br>IFN-r (XMG1.2) Cat# 505810: https://www.biolegend.com/en-us/products/apc-anti-mouse-ifn-gamma-antibody-993?GroupID=GROUP24<br>CD86(GL-1) Cat# 105030: https://www.biolegend.com/en-us/search-results/apc-cyanine7-anti-mouse-cd86-antibody-6554<br>CD40 (3/32) Cat# 124610: https://www.biolegend.com/en-us/products/pe-anti-mouse-cd40-antibody-4983?Clone=3/23<br>CD16/CD32 (93) Cat# 14-0161-82: https://www.thermofisher.com/antibody/product/CD16-CD32-Antibody-clone-93-Monoclonal/14-0161-82#:~:text=CD16%20(Fc%20gamma%20III%20Receptor,use%20in%20flow%20cytometric%20analysis.<br>The specificities of listed imaging antibodies have been validated by the manufacturer by immunofluorescence.<br>anti-GFP (Abcam ab13970): https://www.abcam.com/gfp-antibody-ab13970.html<br>anti-Flag (Sigma F3165): https://www.sigmaaldrich.com/US/en/product/sigma/f3165<br>anti-mcherry (ThermoFisher M11217): https://www.thermofisher.com/antibody/product/mCherry-Antibody-clone-16D7-Monoclonal/M11217<br>anti-cGAS (Abcam ab252416): https://www.abcam.com/products/primary-antibodies/cgas-antibody-epr23611-101-ab252416.html<br>Goat anti-Chicken IgY (H+L) Secondary Antibody, Alexa Fluor 488: https://www.thermofisher.com/antibody/product/Goat-anti-Chicken-IgY-H-L-Secondary-Antibody-Polyclonal/A-11039<br>Goat anti-Rat IgG (H+L) Cross-Adsorbed Secondary Antibody, Alexa Fluor 594: https://www.thermofisher.com/antibody/product/Goat-anti-Rat-IgG-H-L-Cross-Adsorbed-Secondary-Antibody-Polyclonal/A-11007<br>Goat anti-Mouse IgG (H+L) Highly Cross-Adsorbed Secondary Antibody, Alexa Fluor 488: https://www.thermofisher.com/antibody/product/Goat-anti-Mouse-IgG-H-L-Secondary-Antibody-Recombinant-Polyclonal/A28175<br>Goat anti-Mouse IgG (H+L) Highly Cross-Adsorbed Secondary Antibody, Alexa Fluor 647: https://www.thermofisher.com/antibody/product/Goat-anti-Mouse-IgG-H-L-Cross-Adsorbed-Secondary-Antibody-Polyclonal/A-21235<br>The specificities of listed WB antibodies have been validated by the manufacturer by western blot.<br>anti-FLAG (Sigma, F3165, clone: M2): https://www.sigmaaldrich.com/US/en/product/sigma/f3165<br>anti-cGAS (CST, 31659, clone: D3O8O): https://www.cellsignal.com/products/primary-antibodies/cgas-d3o8o-rabbit-mab-mouse-specific/31659<br>anti-Lamin B1 (Sigma, ZRB1143, clone: 6K22): https://www.sigmaaldrich.com/US/en/product/sigma/zrb1143<br>anti-HA (Sigma, H3663, clone: HA-7): https://www.sigmaaldrich.com/US/en/product/sigma/h3663<br>anti-V5 (ThermoFisher, R960-25): https://www.thermofisher.com/antibody/product/V5-Tag-Antibody-Monoclonal/R960-25<br>anti-b-actin (Sigma, A2228): https://www.sigmaaldrich.com/US/en/product/sigma/a2228<br>anti-GAPDH (CST, 2118, clone: 14C10): https://www.cellsignal.com/products/primary-antibodies/gapdh-14c10-rabbit-mab/2118<br>Anti-rabbit HRP-linked IgG antibody (CST 7074): https://www.cellsignal.com/products/secondary-antibodies/anti-rabbit-igg-hrp-linked-antibody/7074<br>Anti-mouse HRP-linked IgG antibody (CST 7076): https://www.cellsignal.com/products/secondary-antibodies/anti-mouse-igg-hrp-linked-antibody/7076<br>Anti-rat HRP-linked IgG antibody (CST 7077): https://www.cellsignal.com/products/secondary-antibodies/anti-rat-igg-hrp-linked-antibody/7077

Eukaryotic cell lines

Policy information about cell lines and Sex and Gender in Research

Cell line source(s)	BSC40, HEK293T, MEF and BHK21 cell lines were purchased from ATCC.
Authentication	Cell lines were not authenticated.
Mycoplasma contamination	All of the cell lines were tested negative for mycoplasma contamination.
Commonly misidentified lines<br>(See ICLAC register)	No commonly misidentified cell lines were used.

Animals and other research organisms

Policy information about studies involving animals; ARRIVE guidelines recommended for reporting animal research, and Sex and Gender in Research

Laboratory animals	C57BL/6 and cGAS-/- mice were purchased from the Jackson Laboratory. STINGGt/Gt mice were generated in the laboratory of Russell Vance (University of California, Berkeley). MDA5-/- mice were generated in Marco Colonna's laboratory (Washington University). Irf3-/- mice were provided by Ruslan Medzhitov (Yale University). Irf7-/- mice were generated by Shizuo Akira (Osaka University). All transgenetic mice are C57BJ/6J background. Female mice between 6-8 weeks old were used. These mice were maintained in the animal facility at the Sloan Kettering Cancer Institute. The holding room was maintained at 12:12-h light:dark cycle with room temperature of 70-72 ˚F and relatively humidity ranging from 30%-70%.
Wild animals	The study did not involve wild animals.
Reporting on sex	Male and female were used as reported. No gender differences were identified in the C57BL/6, cGAS-/-, STINGGt/Gt, MDA5-/-, Irf3-/- and Irf7-/- mice.
Field-collected samples	The study did not involve samples collected from the field.
Ethics oversight	All procedures were performed in strict accordance with the recommendations in the Guide for the Care and Use of Laboratory Animals of the National Institute of Health. The protocol was approved by the Committee on the Ethics of Animal Experiments of Sloan-Kettering Cancer Institute.

Note that full information on the approval of the study protocol must also be provided in the manuscript.

Flow Cytometry

Plots

Confirm that:

☒ The axis labels state the marker and fluorochrome used (e.g. CD4-FITC).

☒ The axis scales are clearly visible. Include numbers along axes only for bottom left plot of group (a 'group' is an analysis of identical markers).

☒ All plots are contour plots with outliers or pseudocolor plots.

☒ A numerical value for number of cells or percentage (with statistics) is provided.

Methodology

Sample preparation	Spleens were collected and processed using the Miltenyi GentleMACS™ Dissociator. Spleen homogenates were incubated with red blood cell lysis buffer on ice for 5 min and then quenched with cold PBS. The cell pellets were resuspended with MACS buffer (Miltenyi Biotec) to generate single cell suspension and then filtered through 70 µm nylon mesh prior to FACS analysis. dLNs were digested with collagenase D (2.5 mg/ml) and DNase (50 µg/ml) at 37ºC for 25 min before filtering through 70-µm cell strainer.
Instrument	LSR Fortessa (BD Biosciences)
Software	Flowjo 10.5.3 (Tree Star)
Cell population abundance	When cells were sorted or enriched, the purity was confirmed by flow cytometry and in each case was above 90% purity.
Gating strategy	Cells were first gated by FSC/SSC. Singlets were gated according to the pattern of FSC-H vs. FSC-A. Positive populations were determined by the specific antibodies, which were distinct from negative populations.

☒ Tick this box to confirm that a figure exemplifying the gating strategy is provided in the Supplementary Information.
